# Computational analysis of multimorbidity between asthma, eczema and rhinitis

**DOI:** 10.1371/journal.pone.0179125

**Published:** 2017-06-09

**Authors:** Daniel Aguilar, Mariona Pinart, Gerard H. Koppelman, Yvan Saeys, Martijn C. Nawijn, Dirkje S. Postma, Mübeccel Akdis, Charles Auffray, Stéphane Ballereau, Marta Benet, Judith García-Aymerich, Juan Ramón González, Stefano Guerra, Thomas Keil, Manolis Kogevinas, Bart Lambrecht, Nathanael Lemonnier, Erik Melen, Jordi Sunyer, Rudolf Valenta, Sergi Valverde, Magnus Wickman, Jean Bousquet, Baldo Oliva, Josep M. Antó

**Affiliations:** 1 ISGlobal, Centre for Research in Environmental Epidemiology (CREAL), Barcelona, Spain; 2 Structural Bioinformatics Group, Departament de Ciencies Experimentals i de la Salut, Universitat Pompeu Fabra, Barcelona, Spain; 3 CIBER Epidemiologia y Salud Pública (CIBERESP), Barcelona, Spain; 4 Institut Municipal d'Investigació Mèdica (IMIM), Barcelona, Spain; 5 University of Groningen, University Medical Center Groningen, Groningen Research Institute for Asthma and COPD, Groningen, The Netherlands; 6 University of Groningen, University Medical Center Groningen, Beatrix Children's Hospital, Department of Pediatric Pulmonology and Pediatric Allergology, Groningen, The Netherlands; 7 Inflammation Research Center, VIB, Ghent, Belgium; 8 Department of Respiratory Medicine, Ghent University Hospital, Ghent, Belgium; 9 University of Groningen, Laboratory of Allergology and Pulmonary Diseases, Department of Pathology and Medical Biology, University Medical Center Groningen, Groningen, The Netherlands; 10 Swiss Institute of Allergy and Asthma Research (SIAF), Davos, Switzerland; 11 Christine Kühne–Center for Allergy Research and Education, Davos, Switzerland; 12 European Institute for Systems Biology and Medicine (EISBM), CNRS, Lyon, France; 13 Arizona Respiratory Center, Tucson, Arizona, United States of America; 14 Institute of Social Medicine, Epidemiology and Health Economics, Charité University Medical Centre, Berlin, Germany; 15 National School of Public Health, Athens, Greece; 16 Department of Pulmonary Medicine, Erasmus MC, Rotterdam, the Netherlands; 17 Institute of Environmental Medicine, Karolinska Institutet, Stockholm, Sweden; 18 Sach's Children's Hospital, Stockholm, Sweden; 19 Division of Immunopathology, Department of Pathophysiology and Allergy Research, Center of Pathophysiology, Infectiology and Immunology, Medical University of Vienna, Vienna, Austria; 20 Christian Doppler Laboratory for Allergy Research, Medical University of Vienna, Vienna, Austria; 21 ICREA-Complex Systems Lab, Universitat Pompeu Fabra, Barcelona, Spain; 22 Institut de Biologia Evolutiva, CSIC-UPF, Barcelona, Spain; 23 Hopital Arnaud de Villeneuve University Hospital and Inserm, Montpellier, France; Cincinnati Children's Hospital Medical Center, UNITED STATES

## Abstract

**Background:**

The mechanisms explaining the co-existence of asthma, eczema and rhinitis (allergic multimorbidity) are largely unknown. We investigated the mechanisms underlying multimorbidity between three main allergic diseases at a molecular level by identifying the proteins and cellular processes that are common to them.

**Methods:**

An *in silico* study based on computational analysis of the topology of the protein interaction network was performed in order to characterize the molecular mechanisms of multimorbidity of asthma, eczema and rhinitis. As a first step, proteins associated to either disease were identified using data mining approaches, and their overlap was calculated. Secondly, a functional interaction network was built, allowing to identify cellular pathways involved in allergic multimorbidity. Finally, a network-based algorithm generated a ranked list of newly predicted multimorbidity-associated proteins.

**Results:**

Asthma, eczema and rhinitis shared a larger number of associated proteins than expected by chance, and their associated proteins exhibited a significant degree of interconnectedness in the interaction network. There were 15 pathways involved in the multimorbidity of asthma, eczema and rhinitis, including *IL4* signaling and *GATA3*-related pathways. A number of proteins potentially associated to these multimorbidity processes were also obtained.

**Conclusions:**

These results strongly support the existence of an allergic multimorbidity cluster between asthma, eczema and rhinitis, and suggest that type 2 signaling pathways represent a relevant multimorbidity mechanism of allergic diseases. Furthermore, we identified new candidates contributing to multimorbidity that may assist in identifying new targets for multimorbid allergic diseases.

## Introduction

During the last years, an increasing attention has been given to multimorbidity as the co-occurrence of two or more medical conditions within a person more often than would be expected by chance [1]. Asthma, eczema and rhinitis (so-called allergic diseases) are complex diseases which tend to co-occur in the same subjects. Children, in particular, frequently present concomitant or consecutive diseases [2,3], something that we refer to as allergic multimorbidity. The MeDALL (Mechanisms of the Development of Allergy) study [4], including both canonical epidemiological methods [5] and unsupervised cluster analysis [6], has shown that coexistence of asthma, eczema and rhinitis in the same child is more common than expected by chance alone, supporting the existence of a multimorbidity cluster. Although IgE sensitization is independently associated with excess multimorbidity of asthma, eczema and rhinitis, its presence accounted only for 38% of multimorbidity [5], suggesting that both IgE and non-IgE mechanisms are involved in the multimorbidity of these diseases [5,6].

Currently, the knowledge about the common mechanisms of allergic multimorbidity relies on few candidate mechanisms. Multiple genes have been identified through linkage and genome wide association studies (GWAS) that contribute to IgE sensitization as well as to asthma, eczema, rhinitis as individual entities. For example, of the ten genome-wide significant loci identified in a large meta-analysis of GWAS on allergic sensitization [7], five were significantly associated with asthma in the GABRIEL consortium asthma meta-analysis [8], but only two were associated with eczema [9]. One of these loci, the *C11orf30-LRRC32* region may represent a common locus for allergic diseases through biological pathways involved in the regulation of IgE, polysensitization, eosinophilic inflammation and co-morbid allergic diseases [10]. Another common locus, ST*AT6*, encodes a transcription factor expressed in B-cells, where it binds to DNA regions stimulating IgE production after B cell activation. In addition to IgE sensitization, many other mechanisms are likely to play a role in the allergic multimorbidity, including prevalent mutations (e.g. filaggrin) and rare genetic variants, structural variants associated to allergic diseases, and epigenetic mechanisms including DNA methylation and miRNA [11].

Despite the evidence of common mechanistic links between asthma, eczema and rhinitis, how these different mechanisms jointly contribute to the allergic multimorbidity is still unclear [10]. MeDALL has also made the hypothesis that allergic multimorbidity and polysensitization are associated with type 2 pathways [11, 12]. Diseases are seldom a consequence of a malfunction or expression change of one single protein, but rather the perturbation of intricate cellular networks, whose diversity and inter-dependence can also affect the activity of genes and proteins functionally linked to other disease-causing genes [13]. Thus, network-based analysis of biological networks, such as the metabolic network and the protein-protein interaction network, can help to identify the mechanisms of multimorbidities [14–16].

Following our findings in MeDALL showing that asthma, eczema and rhinitis in children constitute an allergic multimorbidity cluster suggesting common pathways [5, 6], we undertook an *in silico* study consisting in a computational network-based analysis to identify common proteins among these diseases and their corresponding cellular processes.

## Materials and methods

### Proteins associated to the multimorbidity of asthma, eczema and rhinitis

#### Data sources

We built a set of disease-associated genes from the following databases: Online Mendelian Inheritance in Man (OMIM) [17], ENSEMBL 77 Short Variations [18] databases (via BioMart [19]) and the Comparative Toxicogenomics Database (CTD) [20]. OMIM and CTD provide highly reliable gene-disease associations characterized through various experimental procedures and a process of expert curation of the literature. Since CTD contains a hierarchical collection of diseases, we considered genes associated to any of our diseases of interest and their descendants. Furthermore, only genes labeled as *marker* (i.e. experimentally associated with a disease) in the CTD database were used. Ensembl Variation contains curated information on disease-associated genetic variations from linkage data, genome-wide association studies and other genomic sources. Because the use of GWAS-derived data as a means for characterization of risk factors is debated [21–23], we wished to evaluate the influence that GWAS-derived data might have on our results. Thus, we repeated our analysis with a set of disease-associated gene set that excludes data derived solely from GWAS studies (S1 Text). Because the aim of our study was to explore the mechanisms of the diseases in the framework of the cellular network of protein interactions, the proteins encoded by the disease-associated genes were obtained in UniProt nomenclature [24]. Thus, only gene products known to exist *at protein level* (as defined by the UniProt database) were considered. Details on the databases and on the characterization of the gene-disease associations can be found in S1 Table and S2 Text.

#### Fraction of common proteins

The fraction of proteins associated to a pair of diseases was calculated as a classical Jaccard index [25]:

$$f_{dis\text{1}dis\text{2}}=\frac{\left|N_{dis\text{1}}\cap N_{dis\text{2}}\right|}{\left|N_{dis\text{1}}\cup N_{dis\text{2}}\right|}$$

where |N_*dis1*_ ∩ N_*dis2*_| is the number of proteins common to disease 1 and disease 2, and is the number |N_*dis1*_ U N_*dis2*_| of distinct proteins in disease 1 and disease 2. Similarly, the Jaccard index for a triad of diseases was calculated as:

$$f_{dis\text{1}dis\text{2}}=\frac{\left|N_{dis\text{1}}\cap N_{dis\text{2}}\cap N_{dis\text{3}}\right|}{\left|N_{dis\text{1}}\cup N_{dis\text{2}}\cup N_{dis\text{3}}\right|}$$

where |N_*dis1*_ ∩ N_*dis2*_ *∩ N*_*dis3*_| is the number of proteins common to disease 1, disease 2 and disease 3, and |N_*dis1*_ U N_*dis2*_ U N_*dis3*_| is the number of distinct proteins in disease 1, disease 2 and disease 3.

#### Validation models

We devised two models to test the significance for the observed number of proteins common to any combination of asthma, eczema and rhinitis. In the first one, protein-disease associations were randomized from all proteins in the proteome (14,754) to generate a null distribution [26, 27]. This model tested whether the fraction of common proteins was higher than random expectation, and will be simply referred to as *random model*. We generated 10^3^ instances of this model, and the statistical significance was assessed by means of a *z*-test. The second model tested if the observed fraction of common proteins was significantly higher than expected for any pair or trio of randomly chosen diseases belonging to the *Immune System Diseases* category of the CTD database. We generated 10^3^ pairs and trios of these diseases avoiding the grouping of diseases that are subtypes (or *descendants*, in CTD terminology) of one another. The list of the selected immune system diseases and their associated proteins can be found in S2 Table. The list of all random pairs and trios of immune system diseases is available as S1 File. Because of the skewness in the distribution of the fraction of common proteins in the random models, the statistical significance was assessed by means of a comparison to the empirical distribution. All calculations in this study were performed with the R statistical software [28].

### Network connectivity between asthma, eczema and rhinitis

#### Functional Interaction Network

In order to identify interactions between proteins associated to asthma, eczema and rhinitis, we built the functional interaction network (FIN). The FIN was obtained by combining data from: (1) the Reactome Functional Interaction Network (v. 2013), which contains pairwise protein interactions of different nature such as protein-protein interactions, gene expression interaction, metabolic interactions and signal transduction [29] (interactions annotated as *predicted* were discarded, as were those with score ≤ 0.5, as suggested by the authors); (2) the HIPPIE network, which integrates multiple experimental protein-protein interaction datasets [30] (only HIPPIE interactions scoring > 0.73 were considered, as they are accounted as *high confidence* by the authors); and (3) the innateDB database, which maintains a curated collection of protein-protein interaction data centered on innate immunity proteins [31]. We selected only those interactions from innatedDB with *hpr* < 20 and *lpr* < 20 [32, 33]. The FIN was graphically represented with the Cytoscape software [34].

#### Connectivity assessment

In order to assess if proteins common to any combination for asthma, eczema and rhinitis showed a larger connectivity in the FIN than expected by chance, we used the topological overlap, a generalized metric designed to measure the connectivity between two nodes in terms of the commonality of the nodes that they connect to [35, 36]. For any pair of proteins in the FIN, the topological overlap ranges from 0 (meaning absence of connectivity) to 1 (meaning maximal possible connectivity). The topological overlap (TO) between proteins *a* and *b* is defined as:

$$TO_{a,b}=\frac{\left|N_{a}\cap N_{b}\right|}{\text{min}\left(\left|N_{a}\right|,\left|N_{b}\right|\right)}$$

where |N_*a*_ ∩ N_*b*_| is the number of neighbors common to *a* and *b* (plus 1 if they are directly connected), and min(|N_*a*_|, |N_*b*_|) is the smaller of the |N_*a*_| and |N_*b*_| degrees. TO_a,b_ = 0 if there is no connection between the *a* and *b* and if they do not share any neighbors. TO_a,b_ = 1 if one set of neighbors is a subset of the other (although *a* and *b* may not be directly connected). We calculated the mean TO for proteins: a) common to the diseases under study, and b) unique to the diseases under study. An example of the calculation of the topological overlap can be found in S1 and S2 Figs.

#### Validation models

To obtain the random expectation for the topological overlap, we generated a null distribution based on 10^3^ models where the disease-associated proteins were randomly exchanged by other proteins in the FIN, while keeping constant the number of proteins shared by the diseases and making sure that the exchanged proteins belonged in the same connectivity bin, so as to approximately keep the connectivity. The connectivity bin for a node of degree in the network *d* was calculated as *round(ln(d)+1)* [37]. This model tested whether the observed connectivity was higher than random expectation. The second model tested if the observed number of common proteins was significantly higher than expected for any pair or trio of randomly chosen immune system diseases. We selected immune system diseases as described in the previous section, adding the condition that at least one of their associated proteins should be present in the FIN. We generated 10^3^ instances of each model. The statistical significance was tested as described in the previous section.

### Cellular pathways shared between diseases

Information on the involvement of the proteins in cellular pathways was obtained from the BioCarta database [38] and mapped onto the FIN. The number of cellular pathways with more than 2 edges in the FIN was 273. We measured the fraction of edges rather than the fraction of nodes (i.e. proteins) because we considered that edges better represented the influence of the network connectivity. We calculated how similarly two diseases *dis1* and *dis2* perturbed a cellular pathway *p* by means of a Jaccard index, which we called *Functional Similarity* (FSim):

$$FSim^{p}{}_{dis\text{1}dis\text{2}}=\frac{\left|e^{p}{}_{dis\text{1}}\cap e^{p}{}_{dis\text{2}}\right|}{\left|e^{p}{}_{dis\text{1}}\cup e^{p}{}_{dis\text{2}}\right|}$$

where e^*p*^_*dis1*_ is the number of edges associated to the pathway *p* and disease 1, and e^*p*^_*dis2*_ is the number of edges associated to the pathway *p* and disease 2. For three diseases, the Jaccard Index was defined as:

$$FSim^{p}{}_{dis1dis2dis3}=\frac{\left|e^{p}{}_{dis\text{1}}\cap e^{p}{}_{dis\text{2}}\cap e^{p}{}_{dis3}\right|}{\left|e^{p}{}_{dis\text{1}}\cup e^{p}{}_{dis\text{2}}\cup e^{p}{}_{dis3}\right|}$$

Where e^*p*^_*dis3*_ is the number of edges associated to the pathway *p* and disease 3. An edge was considered associated to a disease if at least one of its nodes was associated to it. In order to avoid some pathways spanning throughout most of the FIN due to the presence of highly connected nodes, we considered an edge as associated to a cellular pathway only if both nodes were associated to it. The proteins associated to the cellular pathways used in this study, together with their interconnections in the FIN, are provided in S3 Table. For each pathway, this process gave us a distribution of observed *Fsim* values, which was tested against random expectation (using the model described in the previous section) and against the distribution obtained for random pairs/trios of randomly selected immune system diseases. Benjamini-Hochberg correction was used to account for multiple testing [39].

### Predicting multimorbidity-associated proteins

In the previous steps, we described the mechanisms of multimorbidity of asthma, eczema and rhinitis based on known protein-disease associations. In this section, we wished to use the information contained in the FIN to predict novel proteins potentially involved in the multimorbidity process. To do so, we employed the NetZcore algorithm of the GUILD package [40]. This algorithm requires an initial group of nodes, called *seeds*, to be weighted with a score, which will be iteratively assigned to those nodes in the network depending on their connectivity. We generated a scored list of potential disease-associated proteins for each disease independently, using a seed score = 1 for known disease-associated proteins, and 0 otherwise. In order to compute *z*-scores, 10^3^ random networks were generated by randomly exchanging edges between pairs of nodes [41].

The resulting lists of candidate proteins were compared to proteins suggested to be related to multimorbidity in the literature. We used the text-mining tool Génie to extract gene names from PubMed abstracts related to multimorbidity between the diseases included in this study [42]. Génie relies on NCBIs curated associations between MedLine records and unambiguous gene identifiers, and employs a Bayesian classifier to associate them to the user’s query terms, outperforming similar text-mining tools (S2 Text for details). The complete set of keywords that we used to retrieve multimorbidity-related proteins (and the Génie parameters for the queries) can be found in S4 Table. The complete list of multimorbidity-associated proteins returned by Génie can be found in S5 Table. Because PubMed abstracts may contain predicted protein-disease associations, we excluded any abstract containing the words *predicted* or *prediction*. We also checked that we were not excluding abstracts labelling genes as *predictors* or mentioning genes *with predictive value*, which could have resulted in false negatives (S2 Text, S6 Table). A Fisher's Exact Test was used to test the statistical association between our predictions and multimorbidity-related proteins obtained by Génie. Since scientific articles about comorbid diseases may not actually employ the term “comorbidity” in the abstract, we carried out a supplementary analysis were the words “comorbidity” and “comorbid” were not present in the PubMed search (see S7 Table for the results).

## Results

### Proteins associated to the multimorbidity of asthma, eczema and rhinitis

**Fig 1:**
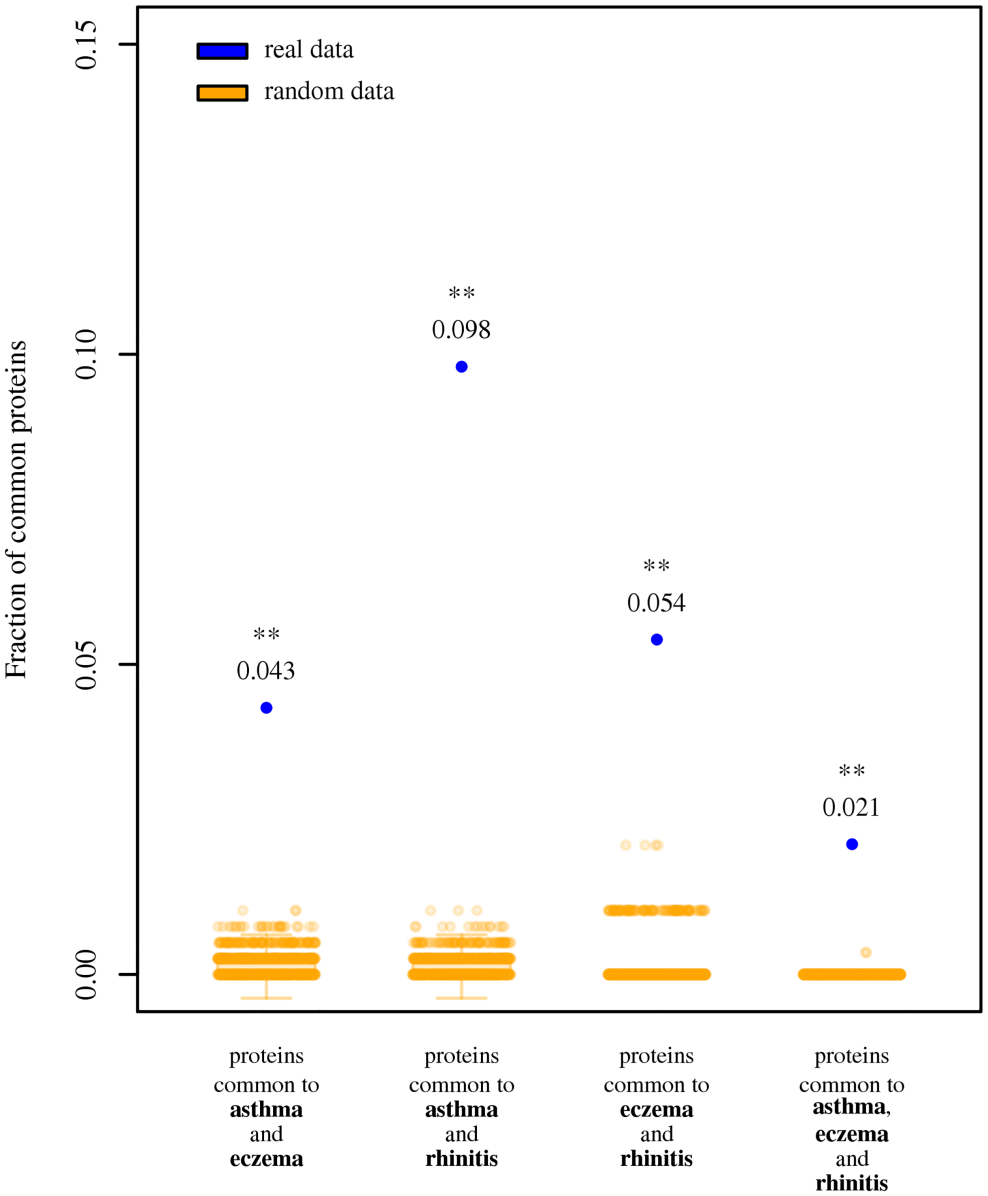
Fraction of proteins associated to asthma, eczema and rhinitis. Blue dots indicate the observed fraction of proteins. Orange scatter boxplots indicate random expectation. One asterisk: observed results are significantly larger than random expectation (z-test; P < 0.05). Two asterisks: observed results are significantly larger than random expectation (z-test; P < 0.01).

We observed 196 proteins associated to asthma, 49 to eczema, and 40 to rhinitis. S8 Table contains the list of all disease-associated proteins. S9 Table contains all disease-associated proteins for associations excluding GWAS-derived data. The number of proteins unique to one disease was 150 for asthma, 44 for eczema and 38 for rhinitis. The list of proteins common to any combination of diseases can be found in Table 1. The three pairs of diseases and the triad shared more proteins than could be expected by random chance (*z*-test; *P <* 0.01 in all cases; Fig 1; S3 Fig for absolute counts; S1 Text and S4 Fig for results excluding GWAS-derived data). Five proteins (*IL4*, *IL13*, *IL1RL1*, *IL18R1* and *TSLP*) were common to all three diseases (*z*-test; *P <* 0.01 in all cases). Furthermore, they also shared a significantly larger fraction of proteins than observed for pairs and triads of randomly chosen immune system diseases (S5 Fig; empirical distribution test; *P <* 0.01 in all cases).

**Table 1:** List of proteins associated to at least two diseases. The complete list of all disease-associated proteins can be found at S8 Table. Number of proteins associated to asthma and eczema: 16; to asthma and rhinitis: 35; to eczema and rhinitis: 5. To all three diseases: 5.

Protein name (UniProt accession)	gene name (HGNC)	protein name	location	asthma	eczema	rhinitis
P05112	*IL4*	interleukin 4	5q31.1	√	√	√
P35225	*IL13*	interleukin 13	5q31.1	√	√	√
Q01638	*IL1RL1*	interleukin 1 receptor-like 1	2q12.1	√	√	√
Q13478	*IL18R1*	interleukin 18 receptor 1	2q12.1	√	√	√
Q969D9	*TSLP*	thymic stromal lymphopoietin	5q22.1	√	√	√
O95760	*IL33*	interleukin 33	9p24.1	√	√	
P01584	*IL1B*	interleukin 1 beta	2q14.1	√	√	
P05113	*IL5*	interleukin 5	5q31.1	√	√	
P13501	*CCL5*	chemokine (C-C motif) ligand 5	17q12	√	√	
P40425	*PBX2*	pre-B-cell leukemia homeobox 2	6p21.32	√	√	
P51671	*CCL11*	chemokine (C-C motif) ligand 11	17q12	√	√	
P51677	*CCR3*	chemokine (C-C motif) receptor 3	3p21.31	√	√	
Q8IZI9	*IFNL3*	interferon, lambda 3	19q13.2	√	√	
Q99466	*NOTCH4*	notch 4	6p21.32	√	√	
Q9Y496	*KIF3A*	kinesin family member 3A	5q31.1	√	√	
Q9Y4H4	*GPSM3*	G-protein signaling modulator 3	6p21.32	√	√	
P01303	*NPY*	neuropeptide Y	7p15.3	√		√
P01906	*HLA-DQA2*	major histocompatibility complex, class II, DQ alpha 2	6p21.32	√		√
P01909	*HLA-DQA1*	major histocompatibility complex, class II, DQ alpha 1	6p21.32	√		√
P01912	*HLA-DRB1*	major histocompatibility complex, class II, DR beta 1	6p21.32	√		√
P01920	*HLA-DQB1*	major histocompatibility complex, class II, DQ beta 1	6p21.32	√		√
P13760	*HLA-DRB1*	major histocompatibility complex, class II, DR beta 1	6p21.32	√		√
P13761	*HLA-DRB1*	major histocompatibility complex, class II, DR beta 1	6p21.32	√		√
P16109	*SELP*	selectin P	1q24.2	√		√
P20039	*HLA-DRB1*	major histocompatibility complex, class II, DR beta 1	6p21.32	√		√
P21731	*TBXA2R*	thromboxane A2 receptor	19p13.3	√		√
P24394	*IL4R*	interleukin 4 receptor	16p12.1	√		√
P50135	*HNMT*	histamine N-methyltransferase	2q22.1	√		√
P84022	*SMAD3*	SMAD family member 3	15q22.33	√		√
Q13093	*PLA2G7*	phospholipase A2 group VII	6p12.3	√		√
Q15399	*TLR1*	toll-like receptor 1	4p14	√		√
Q30134	*HLA-DRB1*	major histocompatibility complex, class II, DR beta 1	6p21.32	√		√
Q30167	*HLA-DRB1*	major histocompatibility complex, class II, DR beta 1	6p21.32	√		√
Q5Y7A7	*HLA-DRB1*	major histocompatibility complex, class II, DR beta 1	6p21.32	√		√
Q8NI36	*WDR36*	WD repeat domain 36	5q22.1	√		√
Q95IE3	*HLA-DRB1*	major histocompatibility complex, class II, DR beta 1	6p21.32	√		√
Q96D42	*HAVCR1*	hepatitis A virus cellular receptor 1	5q33.3	√		√
Q96QA5	*GSDMA*	gasdermin A	17q21.1	√		√
Q9GIY3	*HLA-DRB1*	major histocompatibility complex, class II, DR beta 1	6p21.32	√		√
Q9HBE5	*IL21R*	interleukin 21 receptor	16p12.1	√		√
Q9HBL0	*TNS1*	tensin 1	2q35	√		√
Q9NQ38	*SPINK5*	serine peptidase inhibitor, Kazal type 5	5q32	√		√
Q9TQE0	*HLA-DRB1*	major histocompatibility complex, class II, DR beta 1	6p21.32	√		√
Q9UIL8	*PHF11*	PHD finger protein 11	13q14.2	√		√
Q9UKT9	*IKZF3*	IKAROS family zinc finger 3	17q21.1	√		√
Q9UKW4	*VAV3*	vav guanine nucleotide exchange factor 3	1p13.3	√		√

### Network connectivity between asthma, eczema and rhinitis

**Fig 2:**
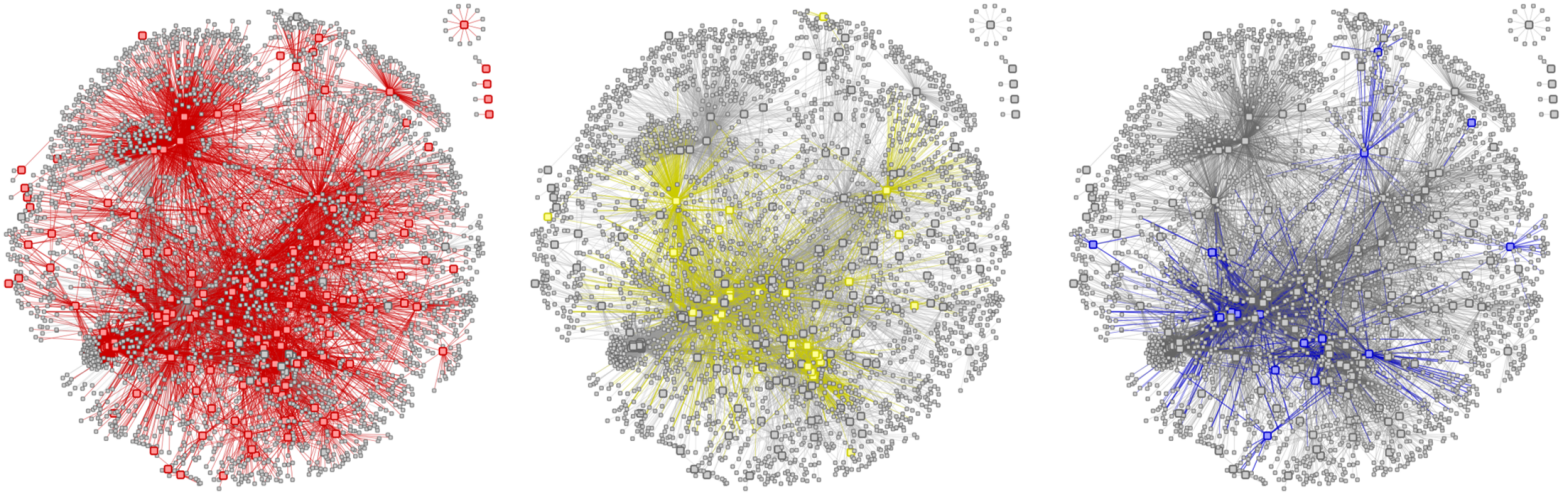
Functional Interaction Networks of asthma, eczema and rhinitis. Fraction of the Functional Interaction Networks comprising the proteins associated to asthma, eczema, rhinitis and all proteins connected to them (i.e. their direct neighbors in the network). A node represents a protein. A link between two nodes represents a functional connection between them. Isolated nodes represent proteins not directly connected neither to any other disease-associated protein nor to any of its direct neighbors. (A) Large red nodes represent asthma-associated proteins. Red links represent functional connections of these proteins. (B) Large yellow nodes represent eczema-associated proteins. Yellow links represent functional connections of these proteins. (C) Large blue nodes represent rhinitis-associated proteins. Blue links represent functional connections of these proteins.

**Fig 3:**
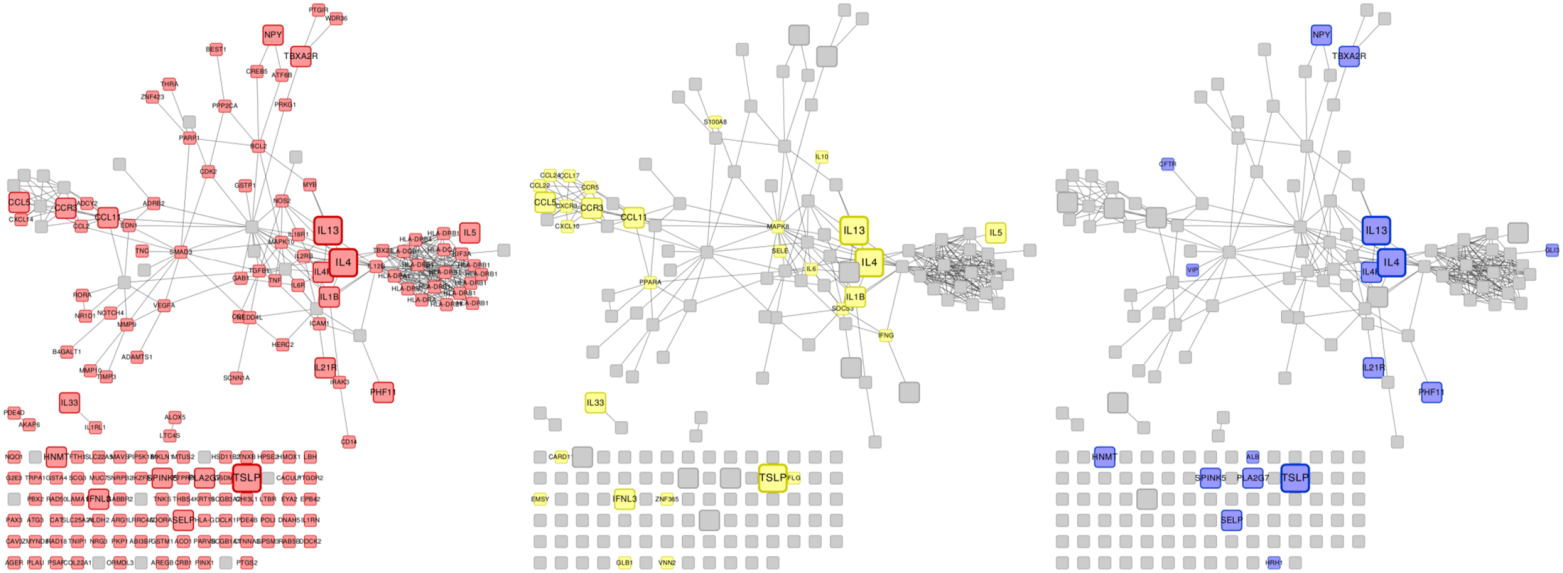
Functional Interaction Networks of asthma, eczema and rhinitis. Fraction of the Functional Interaction Networks comprising the proteins associated to asthma, eczema and rhinitis. A node represents a protein. The size of the node represents the number of disease associations: the large nodes are associated to all diseases, the medium nodes are associated to two diseases, and the small nodes are associated to one disease. A link between two nodes represents a functional connection between them. These networks are a subset of the networks shown in Fig 2, where the direct neighbors have been removed. Isolated nodes at the bottom represent proteins not connected to any protein associated to asthma, eczema and rhinitis. (A) Red nodes represent asthma-associated proteins. (B) Yellow nodes represent eczema-associated proteins. (C) Blue nodes represent rhinitis-associated proteins.

Functional Interaction Network (FIN) contained 9,847 proteins (nodes) involved in 154,164 functional interactions (edges), including 134 immune-related proteins. As not all the proteins associated to the three diseases had highly reliable experimentally-characterized interactions, the number of disease-associated proteins in the FIN was 163 for asthma, 43 for eczema and 32 for rhinitis. Fig 2 shows the portion of the FIN comprising proteins associated to the three diseases and their first-order neighbors (i.e. nodes directly connected to them). Fig 3A, 3B and 3C show the subnetworks for proteins associated to each disease. These figures also provide a visual representation of the pattern of interaction between disease-associate proteins in the FIN, and about what parts of the network are shared between diseases. The complete FIN is available in S2 File. The number of edges between proteins unique to each disease was lower than random chance for all pairs of diseases and for the triad (z-test; *P* < 0.01; S10 Table). We observed that almost all edges belonging to rhinitis-associated proteins are shared with asthma (only 5.4% of edges remain unique to rhinitis), a large overlap that is not seen in any other combination of diseases.

**Fig 4:**
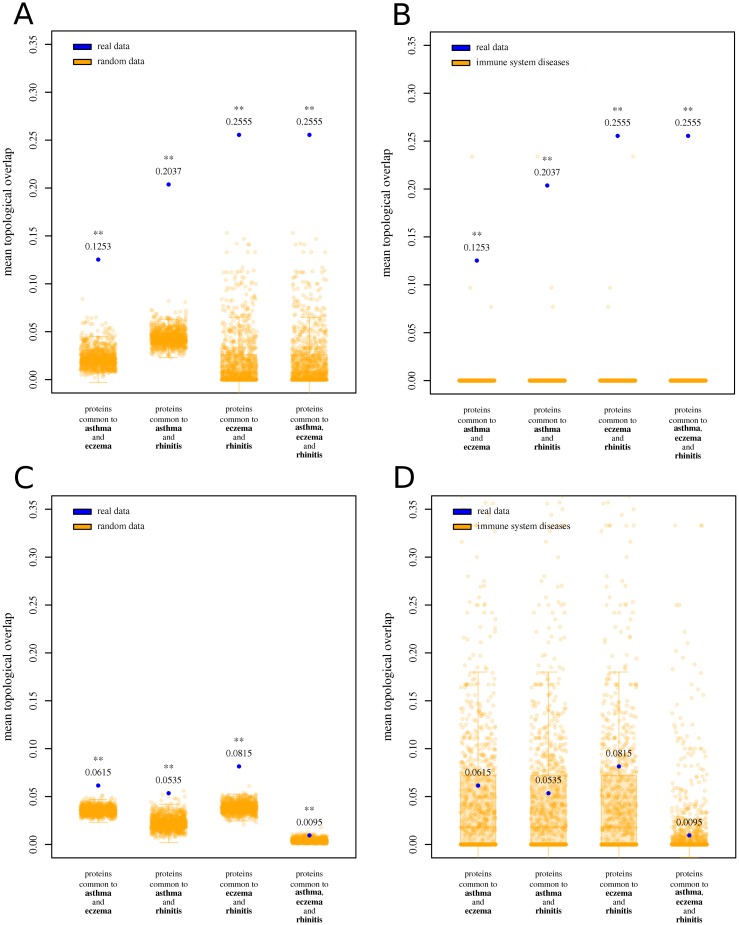
Mean topological overlap for proteins associated to asthma, eczema and rhinitis. (A) Blue dots indicate the observed mean topological overlap (TO) for proteins common to asthma and eczema, asthma and rhinitis, eczema and rhinitis, and common to all three. Orange scatter boxplots indicate random expectation. (B) Blue dots indicate the observed mean TO for proteins common to the combinations of diseases shown in the previous figure. Orange scatter boxplots indicate observed TO values for pairs/trios of immune system diseases. (C) Blue dots indicate the observed mean TO between proteins unique to asthma and unique to eczema, unique to asthma and unique to rhinitis, unique to eczema and unique to rhinitis, and unique to each disease. Orange scatter boxplots indicate random expectation. (D) Blue dots indicate the observed mean TO for proteins unique to the combinations of diseases shown in the previous figure. Orange scatter boxplots indicate observed TO values for pairs/trios of immune system diseases. One asterisk: observed results are significantly larger than random expectation (*P* < 0.05). Two asterisks: observed results are significantly larger than random expectation (*P* < 0.01).

We captured the network-based relationship between the diseases under study with the topological overlap (TO). The TO between proteins common to any combination of diseases was significantly larger than random expectation, and also significantly larger than expected for random pairs/triads of immune-related diseases (z-test; *P* < 0.01 in both cases; Fig 4A and 4B). The same was true for proteins common to all three diseases (z-test; *P* < 0.01; Fig 4A and 4B). However, the TO between proteins unique to any combination of diseases, although significantly larger than random expectation (z-test; *P* < 0.01), was not significantly larger than that observed for random pairs/trios of immune-related diseases (Fig 4C and 4D). S6 Fig shows that proteins unique to each disease are more interconnected than random expectation for asthma, eczema and rhinitis, multimorbidity notwithstanding. For results excluding GWAS-derived data, see S1 Text and S7 Fig.

### Cellular pathways shared between diseases

The origin of any disease can be traced to the perturbation of one or more cellular pathways. One common feature amongst comorbid diseases is that they share a common functionality, because the disruption of a multi-protein pathway can give rise to a number of diseases even if they do not have genes in common [43, 44]. We wished to identify individual pathways that could be linked to the multimorbidity between asthma, eczema and rhinitis, assuming that cellular pathways significantly perturbed by two or three of the diseases are more likely candidate mechanisms for multimorbidity. Fifteen cellular pathways revealed a significant functional similarity for distinct combinations of the three diseases (Table 2). The mechanisms of asthma and eczema were found to be identical for three pathways: *Regulation of hematopoiesis by cytokines*, *GATA3 participate in activating the Th2 cytokine genes expression*, and *CCR3 signaling in eosinophils*. The mechanisms of asthma and rhinitis were identical for the *IL4 signaling pathway* and the *4-1BB-dependent immune response* pathways. Eczema and rhinitis did not show a complete functional similarity for any pathway, being *GATA3 participate in activating the Th2 cytokine genes expression* the pathway that both diseases share with the largest overlap (*Fsim* = 0.67). Other pathways were shared by pairs of diseases at different (but significant) degrees, of which two were exclusive of asthma and eczema: *The role of eosinophils in the chemokine network of allergy*, and *Erythrocyte differentiation pathway*. As for the triad of diseases, no pathway showed a perfected overlap (*Fsim* = 1). This indicates that there is no pathway (or part thereof) identically affected in all three diseases. However, two pathways show a significant three-way overlap: *IL4 signaling pathway* and *GATA3 participate in activating the Th2 cytokine genes expression*. S11 Table contains the functional similarity scores obtained excluding GWAS-derived data, which are discussed in S1 Text. Furthermore, all these observations were significantly larger than expected for pairs and trios of other immune-related diseases (empirical distribution test; *P* < 0.01).

**Table 2:** Functional similarity between asthma, eczema and rhinitis. Numerical values show how similar is the use of a cellular pathway by pairs of trios of diseases. Similarity = 1 means that the diseases affect the pathway in exactly the same way. Similarity = 0 is represented by blank cells. Two asterisks: similarity is significantly larger than random expectation (*z*-test; *P* < 0.01). One asterisk: similarity is significantly larger than random expectation (*z*-test; *P* < 0.05). All significant similarities were also significantly larger than observed for pairs and trios of immune system diseases (empirical distribution test; *P* < 0.01).

Cellular pathway	Overlap score for:			
	asthma and eczema	asthma and rhinitis	eczema and rhinitis	asthma, eczema and rhinitis
Regulation of hematopoiesis by cytokines	1.00 **			
CCR3 signaling in Eosinophils	1.00 **			
The Role of Eosinophils in the Chemokine Network of Allergy	0.57 **	0.14		
IL 4 signaling pathway	0.53 **	1.00 **	0.53 **	0.53 **
GATA3 participate in activating the Th2 cytokine genes expression	1.00 **	0.67 *	0.67 **	0.67 **
Cytokine Network	0.50 *	0.33	0.50 **	0.30 **
IL12 and Stat4 Dependent Signaling Pathway in Th1 Development	0.28	0.40	0.33 **	0.23 **
Th1/Th2 Differentiation	0.25	0.48	0.42 **	0.23 *
Erythrocyte Differentiation Pathway	0.50 *			
The 4-1BB-dependent immune response	0.22	1.00 *	0.22 **	0.22 **
Dendritic cells in regulating TH1 and TH2 Development	0.56 **	0.25	0.33 **	0.22 *
Role of Tob in T-cell activation	0.07	0.57	0.13	0.07
Antigen Dependent B Cell Activation	0.06	0.31	0.20 **	0.06
Cytokines and Inflammatory Response	0.23	0.24	0.25 *	0.10
IL2 Receptor Beta Chain in T cell Activation	0.03	0.27	0.04	0.02
Selective expression of chemokine receptors during T-cell polarization	0.41	0.58	0.37 **	0.30 **
IL 5 Signaling Pathway	0.30	0.30	0.20 *	0.10
NFkB activation by Nontypeable Hemophilus influenzae	0.25	0.25		
TGF beta signaling pathway		0.58		
Adhesion and Diapedesis of Granulocytes	0.11	0.33		
Cell Cycle: G1/S Check Point		0.42		
Monocyte and its Surface Molecules		0.40		
Antigen Processing and Presentation		0.33		
Cells and Molecules involved in local acute inflammatory response		0.29		
Bystander B Cell Activation		0.27		
Activation of Csk by cAMP-dependent Protein Kinase Inhibits. . .		0.26		
Lck and Fyn tyrosine kinases in initiation of TCR Activation		0.26		
The Co-Stimulatory Signal During T-cell Activation		0.26		
Signal transduction through IL1R	0.25			
B Lymphocyte Cell Surface Molecules		0.23		
IL10 Anti-inflammatory Signaling Pathway	0.10			
Cystic Fibrosis Transmembrane Conductance Regulator And Beta 2. . .		0.08		
Pertussis toxin-insensitive CCR5 Signaling in Macrophage	0.07			
Role of ERBB2 in Signal Transduction and Oncology	0.05			
IL 6 signaling pathway	0.05			
Ceramide Signaling Pathway	0.04			
NO2-dependent IL 12 Pathway in NK cells	0.04			
NFAT and Hypertrophy of the heart (Transcription in the broken heart)	0.04			
Signaling of Hepatocyte Growth Factor Receptor	0.03			
TNFR1 Signaling Pathway	0.03			
HIV-I Nef: negative effector of Fas and TNF	0.03			
IL 2 signaling pathway	0.03			
TNF/Stress Related Signaling	0.03			
Keratinocyte Differentiation	0.03			
MAPKinase Signaling Pathway	0.02			

### Predicting multimorbidity-associated proteins

The 30 top-scoring protein candidates that are common to more than one disease are shown in Table 3. The complete list of candidates is shown in S12 Table (and in S13 Table for results excluding GWAS-derived data; see S1 Text for discussion). Although no gold-standard set for multimobidity-related proteins exists for the diseases under study, we observed a clear statistical association between our predictions and those extracted from biomedical literature by the computational tool Génie, with a significant *p*-value in all cases (Fisher's Exact test; *P* < 0.01; Table 4).

**Table 3:** Potential disease-associated proteins predicted for asthma, eczema and rhinitis. NetZcore prediction scores are shown as *z*-scores. Proteins are ranked according to their average *z*-score for all diseases. Empty cell: the protein was not predicted to be associated with the disease with *z*-score > 2.31 (corresponding to *P* < 0.01). *Exp*: the protein is experimentally known to be associated to the disease. This table only shows the 30 top-scoring proteins that were found to be associated to more than one disease. The complete list is available in S12 Table.

Protein name (UniProt accession)	gene name (HGNC)	Protein name	z-score asthma	z-score eczema	z-score rhinitis
Q9Y496	*KIF3A*	kinesin family member 3A	*exp*	exp	10.26
P24394	*IL4R*	interleukin 4 receptor	*exp*	4.21	exp
Q9HBE5	*IL21R*	interleukin 21 receptor	*exp*	3.95	exp
Q9UIL8	*PHF11*	PHD finger protein 11	*exp*	2.85	exp
P16278	*GLB1*	galactosidase beta 1	5.56	*exp*	6.03
O95715	*CXCL14*	chemokine (C-X-C motif) ligand 14	*exp*	10.85	
P20036	*HLA-DPA1*	major histocompatibility complex, class II, DP alpha 1	*exp*		7.93
P04440	*HLA-DPB1*	major histocompatibility complex, class II, DP beta 1	*exp*		7.81
P13500	*CCL2*	chemokine (C-C motif) ligand 2	*exp*	7	
P01903	*HLA-DRA*	major histocompatibility complex, class II, DR alpha	*exp*		6.96
Q29974	*HLA-DRB1*	major histocompatibility complex, class II, DR beta 1	*exp*		6.68
P01911	*HLA-DRB1*	major histocompatibility complex, class II, DR beta 1	*exp*		6.68
P14784	*IL2RB*	interleukin 2 receptor subunit beta	*exp*	2.79	2.35
P29460	*IL12B*	interleukin 12B	*exp*		4.3
P08887	*IL6R*	interleukin 6 receptor	*exp*	4.29	
Q8TE73	*DNAH5*	dynein, axonemal, heavy chain 5	*exp*		3.57
P35625	*TIMP3*	TIMP metallopeptidase inhibitor 3	*exp*	2.46	
P17693	*HLA-G*	major histocompatibility complex, class I, G	*exp*		2.37
Q9P0G3	*KLK14*	kallikrein related peptidase 14	12.9	3.78	18.27
Q9Y337	*KLK5*	kallikrein related peptidase 5	9.01	3.64	12.1
P14091	*CTSE*	cathepsin E	11.16		13.22
P53634	*CTSC*	cathepsin C	11.16		13.22
Q9UBX1	*CTSF*	cathepsin F	11.16		13.22
Q99538	*LGMN*	legumain	10.55		12.39
O00287	*RFXAP*	regulatory factor X associated protein	10.46		12.36
P10619	*CTSA*	cathepsin A	10.24		12.06
P43235	*CTSK*	cathepsin K	9.86		11.47
P52732	*KIF11*	kinesin family member 11	9.64		11.55
O15066	*KIF3B*	kinesin family member 3B	9.61		11.51
O14782	*KIF3C*	kinesin family member 3C	9.4		11.28

**Table 4:** Statistical association between predicted multimorbidity-associated proteins and literature predictions. Literature predictions were automatically extracted from PubMed abstracts using the Génie data mining tool. Statistical association was calculated by means of a Fisher's Exact Test. Confidence intervals are shown in parentheses.

	association	
	odds ratio	*P*
Literature predictions for **asthma** and **eczema**	14.09, 95% CI [6.69, 27.47]	7.22·10^−10^
Literature predictions for **asthma** and **rhinitis**	10.22, 95% CI [3.78, 23.73]	1.61·10^−5^
Literature predictions for **eczema** and **rhinitis**	3.78, 95% CI [1.93, 6.81]	0.00011
Literature predictions for **asthma**, **eczema** and **rhinitis**	6.32, 95% CI [1.90, 16.58]	0.0019

## Discussion

In this paper, we presented a strategy to measure multimorbidity between asthma, eczema and rhinitis at cellular network level. Asthma, eczema and rhinitis share a larger number of associated proteins than expected by chance, and exhibit a significant degree of interconnectedness in the functional interaction network. Computational analysis of the network identified 13 cellular pathways as significantly overlapping between pairs of diseases, and thus potentially involved in allergic multimorbidity and polysensitization mechanisms. Three of these pathways were remarkable because they overlapped in all three pairs of diseases as well as in the three diseases simultaneously: *IL2 activation*, *IL4 signaling pathway* and *GATA3 participate in activating the Th2 cytokine genes expression*. Furthermore, the network analysis allowed predicting many additional proteins as new candidates contributing to multimorbidity. This study strongly supports the *a priori* MeDALL hypothesis proposing that in children asthma, eczema and rhinitis co-occur as an allergic multimorbidity cluster [5, 6] and that both IgE and non-IgE related pathways represent common mechanisms of the multimorbidity of allergic diseases [11, 12].

### Strengths and weaknesses

Our study has some important strengths. This is, to our knowledge, the first time that the multimorbidity between asthma, eczema and rhinitis has been systematically explored with a computational approach. Although there are other network-based studies of asthma, underlying mechanisms of allergic multimorbidity were not considered [45, 46]. For our analysis, we integrated abundant curated evidence from different sources, and we relied on experimentally validated protein-disease associations. Because our aim was to provide a synthesis of current biological knowledge in a framework that can be readily interpreted by researchers, we selected a set of well-described cellular pathways. However, we acknowledge that drawback of our method may be that known tools to find biological plausible pathways may today under-represent relevant tissues that are less accessible (e.g. lung tissue with resident cells like epithelial cells) that have an important barrier and immunological function. For the construction of the FIN we used not only protein-protein interaction but also other kinds of protein-protein functional relationships (e.g. signaling, expression regulation, metabolic relationships) allowing the identification of a wide range of mechanisms. Our network-based analysis revealed information on protein interconnectivity and cellular pathways that could not have been uncovered otherwise. Finally, because our approach is not explicitly disease-centered, it can potentially be applied to any other groups of diseases.

On the other hand, our computational approach is subject to potential limitations, the main of which is the completeness and noisiness of the FIN [14, 23, 26]. We chose to consider only the most reliable interactions, which might have resulted in removing potentially true interactions (false negatives). Also, despite selecting only high-confidence interactions, some false interactions might have been included in this study (false positives). Furthermore, it is worth mentioning that, for simplicity, our network is undirected and independent of differential expression patterns or external factors (e.g. allergens). However, some studies have shown that the current interaction databases do allow the systematic investigation of disease mechanisms [23]. Although we applied the usual procedure of multiple testing correction in our statistical analysis to avoid false positives (type I errors), some authors have argued against its use on the grounds that these corrections inflate the number of false negatives (type II errors) [47]. Finally, it must be taken into account that our gene and protein selection is subject to some restrictions. First, we selected only curated disease-protein associations from several databases, so the quality of our dataset depends on their criteria as to establish what disease-protein associations are the most reliable. Consequently, we chose three manually-curated databases (CTD, OMIM and Ensembl Variation) that are widely employed in computational genotype-phenotype association studies. Part of our associations were GWAS-derived, and there is a debate on the use of this kind of data as a means for characterization of risk factors, based mainly on concerns about its statistical processing and the fact that, without additional functional information, it is often difficult in GWAS analysis to implicate a specific gene in a genotype-phenotype association [48]. However, when integrated with other gene-disease association methods, and particularly when applied to pathway and network analysis, it has been shown to increase statistical power and provide valid results. These results give us additional confidence on the soundness of our results. Second, we cannot exclude publication bias, and the fact that the associations between proteins and diseases may be the result of differences in the interest of researchers and funding sources. Third, as most of the studies captured in this computational study have been conducted in affluent countries where atopy is more common, we should be cautious when inferring our findings to populations where atopy plays a less relevant role in asthma, rhinitis and eczema. Finally, asthma in particular is considered an umbrella term encompassing multiple endotypes, which were not studied independently.

In the foreseeable future, improvements in the quality and coverage of protein interactions will allow for more accurate network-based studies. Although we selected proteins experimentally associated to the diseases under study, the shared proteins and mechanisms are *in silico* observations that will need experimental validation. However, some of the described mechanisms are consistent with the literature as described below.

### Proteins associated to the multimorbidity of asthma, eczema and rhinitis

We found that the number of disease-associated proteins shared by asthma, eczema and rhinitis could neither be explained by random chance nor by the fact that all diseases are related to the immune system. *IL4*, *IL13*, *IL1RL1*, *IL18R1* and *TSLP* were the only proteins common to the three diseases, and thus are important candidates to explain allergic multimorbidity and polysensitization. *IL4*, *IL13* and *TSLP* are cytokines that have been proposed to have a role in multimorbidity in non-atopic [49, 50] and atopic diseases [51]. It is known that both *IL4* and *IL13* are involved type 2 responses, in IgE production in asthma, rhinitis [52] and in eczema [53], as well as in the cellular inflammation of the three diseases [54] as well as in the regulation of the epithelial barrier function in the skin [55], the airways [56], and type 2 responses [57]. *L1RL1* is part of the *IL33* receptor complex, which drives TH2 inflammation. It has been associated to asthma [58], and also plays an important role in intermittent allergic rhinitis [59]. *IL33* has been long associated with asthma but also with allergic rhinitis in murine models [60]. Furthermore, The associated region on chromosome 2q12 contains the family of *IL1-r*eceptor genes, *IL1RL1 (IL1* receptor-like 1), *IL18R1 (IL18* receptor 1), and *IL18RAP (IL18* receptor accessory protein). Members of this family are abundantly expressed in the skin, and *IL1RL1 i*s involved in Th2 responses and is important for the pathogenesis of eczema [61, 62]. *TSLP* is an epithelial *IL7*-like cytokine activating Th2 responses [63], and is responsible for a pattern of inflammation suggestive of multimorbidity between asthma and chronic obstructive pulmonary disease [64], and between different types of dermatitis [65]. *IL4*, *IL13*, *IL1RL1*, *IL18R1* and *TSLP* are also the only proteins found to be common to eczema and rhinitis.

Proteins associated to asthma and eczema are all related to immunity (*IL1ß*, *IL5*, *IL33*, C-C motif chemokine 5, eotaxin). *IL18* is also involved in the cell biology of the inflammasome [66]. *IL33* is a crucial regulator of mast cell functions [67] and may be of great importance to understand multimorbidity and polysensitization as it as it modulates the expression of human β-defensin 2 in human primary keratinocytes, and may influence the susceptibility to bacterial superinfection in acute atopic dermatitis [68].

Amongst the proteins common to asthma and rhinitis,there is a number of members of the *HLA-DRB* and *HLD-DQ* families, which have major roles in T-cell activation. HLA plays a role in the development of the IgE response to the allergens, but genetic regulation appears to differ in mono- and polysensitized patients. Associations between HLA haplotypes or HLA-DQ/DR molecules and allergen sensitivity were confirmed only in patients either with low total serum IgE levels or monosensitized [69–71]. The presence of hepatitis A virus cellular receptor 1 (*HAVCR1)* is intriguing, although it may protect against atopy when hepatitis A virus infection rates were high [72]. We also found histamine N-methyltransferase (*HNMT*), a key protein in histidine metabolism, involved in the airways epithelium in asthma and rhinitis [73]. Finally, exclusion of disease-protein associations derived solely from GWAS studies did not noticeably alter our observations (S1 Text).

### Network connectivity between asthma, eczema and rhinitis

Previous studies have concluded that biological network-level links between diseases contribute to the likelihood of individuals developing simultaneous conditions [74]. Visual inspection of Figs 2 and 3 suggests the existence of a region in the functional network is shared by all three diseases. We observed that the connectivity between proteins common to the diseases (measured as the average topological overlap) was significantly larger than random expectation. Although the type 2 response appears to be an important mechanism of multimorbidity and polysensitization, we also observed that the connectivity between proteins shared by asthma, eczema and rhinitis could not be explained solely by the fact that they all are immune-related diseases. Since network connectivity has been largely used as a measure of modularity (and, thus, of mutual functional influence) between proteins [75], these observations imply the existence of a core mechanism specific to the three diseases under study. On the other hand, proteins unique to each disease did not seem to contribute specifically to multimorbidity through their interactions with proteins unique to the other diseases (their modularity was not significantly larger than that of pairs/trios of other immune-related diseases). The analysis of the modularity between proteins unique to each disease suggested the existence of (at least) partial dissociated mechanisms unique to each disease, which would be responsible for their distinct patterns of occurrence and isolated symptomatology. This is supported by the fact that the number of edges between proteins unique to each disease was lower than random chance for all pairs of diseases and for the triad. Also, it has to be noted that, in terms of network functionality, rhinitis shares most of its mechanisms with asthma (a commonality that was not obvious when comparing nodes alone). This relationship has been observed elsewhere [11, 76].

### Cellular pathways shared between diseases

The presence of type 2-related pathways amongst the top-scoring pathways affected by the three diseases supports the influence of the type 2 gene cluster in multimorbidity between asthma and eczema [77]. The pathway *GATA3 participates in activating the Th2 cytokine genes expression* show the highest overlap for the three diseases. Transcription factor *GATA3* is highly expressed in peripheral blood ILC2s cells during inflammatory responses, and is essential for interleukin-4 expression [78]. Recently, therapeutic targeting *GATA3* has proven beneficial in attenuating asthmatic responses [79]. ILC2, a type of innate lymphoid cells producing cytokines such as *IL9*, and *IL13*, plays an important role in eosinophilic asthma [80] in response to respiratory infections [81], and is over-expressed in eczema lesions and in allergic rhinitis subjects [82, 83]. The three diseases also show a significantly high similarity in the use of the pathway *IL 4 signaling pathway* suggesting the existence a core mechanism around ILC2s and *IL4* that connects the mechanisms of the three diseases. It is also noteworthy the presence of two eosinophil-related pathways shared solely by asthma and eczema: *CCR3 signaling in Eosinophils* and *The role of eosinophils in the chemokine network of allergy*. Furthermore, the similarity that we observed in the use of cellular pathways by asthma, eczema and rhinitis could not be explained by their immune-related nature alone, despite the fact that some response mechanisms are shared among immune-related diseases [46]. This suggests that these mechanisms are specific for multimorbidity between the three diseases under study.

### Predicting multimorbidity-associated proteins

We also used the network connectivity measures to predict proteins that could be associated with multimorbidity. Amongst the top-scoring proteins predicted to play a role in a three-way multimorbidity between asthma, eczema and rhinitis we identified interleukin receptors *IL4R*, *IL21R* and *IL2RB*. Both *IL4R* and *IL21R* are strongly related to the immune response, and are already known to be associated to asthma and rhinitis. *IL2RB* is currently associated to asthma and predicted to be associated also to eczema and rhinitis. All these receptors bind relevant cytokines regulating lymphocytic activity. Kallikrein-5 and kallikrein-14 (*KLK5*, *KLK14*) are two other proteins predicted to play a role in a three-way multimorbidity. Both proteins belong to a family of highly specific serine-proteinases whose deregulation is linked to several allergic diseases [84]. Recently, one kallikrein gene family member (*KLKB1*) was found to regulate soluble cleaved urokinase plasminogen activator receptor [85]. *KLKB1* cleaves *UPAR* and negates soluble cleaved *UPAR* effects on primary human bronchial epithelial cells. Interestingly, *scuPAR* is encoded by *UPAR*, a gene found by positional cloning in asthma families [85]. Other proteins predicted to be related to multimorbidity are *KIF3A*, already known to be associated to asthma, eczema and the atopic march [86], and predicted to be implicated also in rhinitis. Interestingly, seven *KIF3A* SNPs were actually reported to be associated with rhinitis in one study that was not incorporated to the databases used in this study [87], thus supporting the reliability of the prediction method. Lastly, *PHF11*, a transcriptional activator of the Th1 effectors interleukin-2 (*IL2*) and interferon-γ (*IFNG*), is predicted to be associated to eczema. The highest-scoring candidate protein for comorbidity between asthma and eczema were C-X-C motif chemokine 14 (*CXCL14*), a known mediator in inflammatory processes, and myeloblastin (*PRTN3*), a matrix-degrading proteinase also related to asthma [88], and chemokine ligand *CCL2*, known to be upregulated by *IL31*, which in turn is one of the main inducers of skin pruritus in eczema [89]. As candidates for comorbidity between asthma and rhinitis, we identified in the top-scoring position several members of the class II MHC, owing probably to the number of proteins of the same family which are already known to be associated to both diseases. Also, we identified several members of the cathepsin family of proteases, known to be involved in many inflammatory processes, amongst them airway inflammation.

It has to be noted, though, that the identification of an association between a protein and a disease depends on the variable criteria used in different databases. It is thus possible that some of the predicted proteins in our study are reported as actually associated to the diseases according to some other databases or studies. Furthermore, the association test performed (Table 4) excluded predicted proteins from PubMed abstracts (see Methods). This minimized the number of false positives when comparing these literature-based protein sets to our predictions. However, because we used a keyword-based search, it is possible that some false positives may have been produced because of the diverse wording employed in abstracts. Also, excluding the words *predicted* and *prediction* (see Methods) might have introduced some false negatives by removing genes

### Applying systems medicine to the understanding of allergic diseases

Asthma, eczema and rhinitis are salient examples of complex multimorbid allergic diseases. In the recent years, the importance of applying systems medicine approaches to unravel the complexity of chronic diseases has been highlighted [23, 46]. A central notion of this approach is that diseases are seldom a consequence of a malfunction or expression change of one single protein, but rather the perturbation of intricate cellular networks [13]. The MeDALL study was purposely designed to apply systems medicine approach to understanding the complexity of allergic diseases. With the present computational analysis of the mechanisms of asthma, eczema and rhinitis, we provided a valuable example of how systems medicine can contribute to the understanding of multimorbidity.

## Conclusions

- Asthma, eczema and rhinitis share a larger number of proteins than expected by chance, as well as a significantly higher degree of interconnectedness in the functional interaction network. The fact that these observations cannot be explained solely by their immune-related nature imply that other interrelated mechanisms are common to the three diseases.
- Type 2 response appears to be an important mechanism of multimorbidity, consistent with our previous finding that multimorbidity and polysensitization are closely associated. However, the presence of non-type-2 cellular pathways and the connectivity between proteins shared by asthma, eczema and rhinitis could not be explained solely by the association of the three diseases to the immune system. This suggests that multimordity results from a wider range of interrelated cellular mechanisms.
- Computational analysis of the network identified 15 cellular pathways potentially involved in allergic multimorbidity mechanisms. Two of these pathways were remarkable because they overlapped in all three diseases: *IL4 signaling pathway* and *GATA3 participate in activating the Th2 cytokine genes expression*.
- Network analysis suggested additional proteins as new candidates contributing to multimorbidity. Those proteins were kallikreins, cathepsins and members of the class II MHC, among others. These results are of importance as they can lead to the assessment of new targets for multimorbid allergic diseases.
- The study results strongly agrees with previous MeDALL findings showing that in children asthma, eczema and rhinitis co-occur as an allergic multimorbidity cluster likely due to common mechanisms.

## Supporting information

S1 Text Computational analysis of multimorbidity between asthma, eczema and rhinitis (excluding GWAS-derived association data). (PDF)

S2 Text Extended methods. (PDF)

S1 Fig Example of the calculation of the topological overlap for proteins associated to two diseases. (A) Disease A (orange ellipse) is associated to 6 proteins: *p1*, *p2*, *p3*, *p4*, *p5*, *p*6. Disease B (blue ellipse) is associated to 6 proteins: *p4*, *p5*, *p6*, *p7*, *p8*, *p9*. Three proteins are common to both diseases (*p4*, *p5*, *p6*; shown in the intersection of both diseases, in blue with orange border). (B) Mapping of the disease-associated proteins on to a dummy network. As in the previous figure, common proteins (*p4*, *p5*, *p6*) are shown in blue with orange border. (C) Calculation of the average topological overlap (TO) between the common proteins. (D) Calculation of the average topological overlap between the proteins unique to each disease.
(PNG)

S2 Fig Example of the calculation of the topological overlap for proteins associated to three diseases. (A) Disease A (orange ellipse) is associated to 7 proteins: *p1*, *p2*, *p3*, *p4*, *p5*, *p*6, p7. Disease B (blue ellipse) is associated to 7 proteins: *p4*, *p5*, *p6*, *p7*, *p8*, *p9*, *p10*. Disease C (purple ellipse) is associated to 5 proteins: *p4*, *p5*, *p6*, *p7*, *p11*. Proteins common to all diseases are *p6* and *p7* (shown as a blue circles with double purple-orange border). Proteins common solely to diseases A and B are *p4* and *p5* (shown as blue circles with orange border). There are no proteins common solely to diseases A and C, neither to diseases B and C. (B) Mapping of the disease-associated proteins on to a dummy network. (C) Calculation of the average topological overlap (TO) between the common proteins. (D) Calculation of the average topological overlap between the proteins unique to each disease.
(PNG)

S3 Fig Number of proteins associated to asthma, eczema and rhinitis. Blue dots indicate the observed fraction of proteins. Orange scatter boxplots indicate random expectation. One asterisk: observed results are significantly larger than random expectation (z-test; *P* < 0.05). Two asterisks: observed results are significantly larger than random expectation (z-test; *P* < 0.01).
(PNG)

S4 Fig Fraction of proteins associated to asthma, eczema and rhinitis (GWAS-derived data excluded). Blue dots indicate the observed fraction of proteins. (A) Orange scatter boxplots indicate random expectation. One asterisk: observed results are significantly larger than random expectation (z-test; P < 0.05). Two asterisks: observed results are significantly larger than random expectation (z-test; P < 0.01). (B) Orange scatter boxplots indicate fraction of associated proteins for pairs/trios of immune system diseases. One asterisk: observed results are significantly larger than random expectation (empirical distribution test; *P* < 0.05). Two asterisks: observed results are significantly larger than random expectation (empirical distribution test; *P* < 0.01).
(PNG)

S5 Fig Fraction of proteins associated to asthma, eczema and rhinitis. Blue dots indicate the observed fraction of proteins. Orange scatter boxplots indicate fraction of associated proteins for pairs/trios of immune system diseases. One asterisk: observed results are significantly larger than random expectation (empirical distribution test; *P* < 0.05). Two asterisks: observed results are significantly larger than random expectation (empirical distribution test; *P* < 0.01).
(PNG)

S6 Fig Mean topological overlap between proteins associated to a single disease only. Blue dots indicate the observed mean topological overlap (TO) between proteins uniquely associated to either asthma, eczema or rhinitis. Orange scatter boxplots indicate random expectation. One asterisk: observed results are significantly larger than random expectation (*P* < 0.05). Two asterisks: observed results are significantly larger than random expectation (*P* < 0.01).
(PNG)

S7 Fig Mean topological overlap for proteins associated to asthma, eczema and rhinitis (GWAS-derived data excluded). (A) Blue dots indicate the observed mean topological overlap (TO) for proteins *common* to combinations of asthma, eczema and rhinitis. Orange scatter boxplots indicate random expectation. (B) Blue dots indicate the observed mean TO for proteins *common* to combinations of asthma, eczema and rhinitis. Orange scatter boxplots indicate observed TO values for pairs/trios of immune system diseases. (C) Blue dots indicate the observed mean TO for proteins *unique* to combinations of asthma, eczema and rhinitis. Orange scatter boxplots indicate random expectation. (D) Blue dots indicate the observed mean TO for proteins *unique* to combinations of asthma, eczema and rhinitis. Orange scatter boxplots indicate observed TO values for pairs/trios of immune system diseases. One asterisk: observed results are significantly larger than random expectation (*P* < 0.05). Two asterisks: observed results are significantly larger than random expectation (*P* < 0.01).
(PNG)

S1 File Random pairs and trios of immune system diseases. Tab-delimited text file. Diseases were extracted from the CTD database (see Methods in the main paper).
(GZ) Click here for additional data file.

S2 File Functional Interaction Network. This network was generated by combining data from the HIPPIE, Reactome and InnateDB databases (see Methods in the main paper).
(GZ) Click here for additional data file.

S1 Table Source of disease-protein associations. OMIM: On-line Mendelian Inheritance in Man; CTD: Comparative Toxicogenomics Database; EV84: Ensembl Variation 84.
(PDF)

S2 Table Diseases in the *immune system diseases* category in CTD database. Disease-associated proteins (provided as UniProt accessions), are separated by a semicolon. The *network* column indicates whether the disease has at least one associated protein present in then FIN (1) or not (0).
(XLS)

S3 Table Pathways in BioCarta database. Pathway-associated proteins (provided as UniProt accessions) and interactions between pathway-associated proteins are separated by a semicolon.
(XLS)

S4 Table Parameters used in the Génie tool. The Génie tool (http://cbdm-01.zdv.uni-mainz.de/~jfontain/cms/*)* was used to extract gene names present in PubMed abstracts related to a topic of interest, as defined by a PubMed query.
(DOC)

S5 Table Multimorbidity-associated genes obtained via the Génie data mining tool. Columns are as follows: Query: PubMed query used; Rank: position (rank) of the gene (ordered by ascending FDR); GeneID: gene name; n: number of PubMed abstracts manually associated to the gene; n_pos: number of PubMed abstracts that Génie has selected at *P* < 0.01; FDR: False Discovery Rate; Top 10 PMIDs: top 10 PMIDs for each gene, together with the associated *p*-value. More information at http://cbdm-01.zdv.uni-mainz.de/~jfontain/cms/.
(XLS)

S6 Table Parameters used in the Génie tool (predicted genes excluded). The Génie tool was used to extract gene names present in PubMed abstracts related to a topic of interest, as defined by a PubMed query. Unlike S4 Table, this table does not exclude the terms *predicted* nor *prediction*, and includes the terms *predictive* or *predictor*.
(DOC)

S7 Table Statistical association between predicted multimorbidity-associated proteins and literature predictions. Literature predictions were automatically extracted from PubMed abstracts using the Génie data mining tool. The terms used to query PubMed database were those shown in S4 Table minus the word “comorbidity”. Statistical association was calculated by means of a Fisher's Exact Test. Confidence intervals are shown in parentheses.
(DOC)

S8 Table Complete list of all proteins associated to asthma, eczema and rhinitis. Gene names are provided as common (HGNC) gene names and protein names as UniProt accessions.
(XLS)

S9 Table Complete list of all proteins associated to asthma, eczema and rhinitis (GWAS-derived data excluded). Gene names are provided as common (HGNC) gene names and protein names as UniProt accessions.
(XLS)

S10 Table Fraction of edges associated to asthma, eczema and rhinitis. Mean random expectation in shown between parenthesis. The fraction of common edges was calculated using the Jaccard index (see Methods in the main paper). The fraction of common edges is statistically larger than random expectation in all four cases (p-value < 0.01). The fraction of unique edges is statistically lower than random expectation in all four cases (p-value < 0.01). The number of edges associated to asthma: 8560; number of edges associated to eczema: 2584; number of edges associated to rhinitis: 2898.
(DOC)

S11 Table Functional similarity between asthma, eczema and rhinitis (GWAS-derived data excluded). Numerical values show how similar is the use of a cellular pathway by pairs of trios of diseases. Similarity = 1 means that the diseases affect the pathway in exactly the same way. Similarity = 0 is represented by blank cells. Dark blue cells: similarity is significantly larger than random expectation (*z*-test; *P* < 0.01). Light blue cells: similarity is significantly larger than random expectation (*z*-test; *P* < 0.05). All significant similarities were also significantly larger than observed for pairs and trios of immune system diseases (empirical distribution test; *P* < 0.01).
(XLS)

S12 Table Complete list of predicted disease-associated proteins. Gene names are provided as common gene names and protein names as UniProt accessions. NetZcore prediction scores are shown as *z*-scores. Proteins are ranked according to their average *z*-score for all diseases. Empty cell: the protein was not predicted to be associated with the disease with *z*-score > 2.31 (corresponding to *P* < 0.01). *Exp*: the protein is experimentally known to be associated to the disease. Proteins predicted (or experimentally known) to be associated to more than one disease are candidates to multimorbidity.
(XLS)

S13 Table Complete list of predicted disease-associated proteins (GWAS-derived data excluded). Gene names are provided as common gene names and protein names as UniProt accessions. NetZcore prediction scores are shown as *z*-scores. Proteins are ranked according to their average *z*-score for all diseases. Empty cell: the protein was not predicted to be associated with the disease with *z*-score > 2.31 (corresponding to *P* < 0.01). *Exp*: the protein is experimentally known to be associated to the disease. Proteins predicted (or experimentally known) to be associated to more than one disease are candidates to multimorbidity.
(XLS)
