# Supplementary material for: Computational analysis of multimorbidity between asthma, eczema and rhinitis
Source: PLoS One. 2017 Jun 9;12(6):e0179125. doi: 10.1371/journal.pone.0179125 (PMC5466323; doi:10.1371/journal.pone.0179125)
Supplement: S1 Text — (PDF) [file pone.0179125.s001.pdf]

TEXT S1. Computational Analysis of Multimorbidity Between Asthma, Eczema and Rhinitis (excluding GWAS-derived association data)

MATERIALS AND METHODS

Proteins associated to the multimorbidity of asthma, eczema and rhinitis

We built the disease-protein associations following the procedures described in the main paper, but excluding disease-protein associations established solely by means of GWAS studies (**Table S9**).

RESULTS AND DISCUSSION

We intended to measure the effect to GWAS-derived data on our results, so we replicated the study excluding explicitly this kind of data. This implies a removal of potentially spurious genotype-phenotype associations at the cost of a reduction in the statistical power of the study (since true associations might also be removed).

Proteins associated to the multimorbidity of asthma, eczema and rhinitis

145 proteins were associated with asthma, 34 with eczema, and 17 with rhinitis. This is a reduction of 51 (26%) proteins for asthma, 15 (30.6%) for eczema and 23 (42.5%) for rhinitis compared to associations including GWAS-derived data. The two pairs of diseases and the triad shared more proteins that could be expected by random chance (z-test; $P < 0.01$ in all cases; **Figure S4**). Two proteins (*IL4*, *IL13*; UniProt: P05112, P35225) were common to all three diseases. As per the other three proteins associated to the three disease, *TSLP* was found only to be associated to asthma and eczema when excluding GWAS data, *IL1RL1* was only associated to asthma, and *IL18R1* was not present in the dataset (being associated to the three diseases solely by GWAS studies).

Network connectivity between asthma, eczema and rhinitis

The number of disease-associated proteins in the Functional Interaction Network (FIN) was 121 for asthma, 31 for eczema and 15 for rhinitis. This is a reduction of 42 (25.8%) proteins for asthma, 12 (27.9%) for eczema and 17 (53.1%) for rhinitis compared to including GWAS-derived data. As observed in the main paper, the average topological overlap between proteins common to any combination of diseases was significantly large than random expectation in all cases (empirical distribution test; $P < 0.01$; **Figure S7**).

Cellular pathways shared between diseases

The number of disease-associated proteins present in the FIN and in BioCarta database (see *Methods* in the main paper) was 121 for asthma, 31 for eczema and 15 for rhinitis. This is a reduction of 9 proteins for asthma, 2 for rhinitis and none for eczema compared to including GWAS data (**Table S11**). Furthermore, 50% of all proteins in the FIN associated via GWAS are connected to at least one protein associated to disease by other experiments.

The similarity of the results obtained with and without GWAS data is remarkable, and can be explained due to the fact that the presence of GWAS-derived protein associations in the functional network was low, and only a fraction of those were further related to cellular pathways. Furthermore, the fact that GWAS-derived proteins in the network were mostly connected to non-GWAS-derived ones further decreases their impact on our results, because our network analysis measures shared connections (edges), not individual proteins (nodes). Noteworthy differences are

the absence of the pathways *IL12 and Stat4 Dependent Signaling Pathway in Th1 Development* and *Th1/Th2 Differentiation* as significantly associated to all three diseases. Conversely the pathway *Role of Tob in T-cell activation* shows a small but significant ($P < 0.05$) similarity for all three diseases.

Predicting multimorbidity-associated proteins

The list of protein candidates that is provided in **Table S13**. The list of top-scoring predicted proteins in similar to that in **Table 3** in the main paper. An interesting difference is that proteins associated to the diseases by GWAS studies (and, consequently, removed in this analysis) are predicted with high scores, which highlights the predictive ability of the Netzscore algorithm. For instance, thymic stromal lymphopoietin (*TSLP*), which is associated to the three diseases in the main paper, is also predicted to be associated to the three diseases in **Table S13**. Interleukin 1 receptor-like 1 (*IL1RL1*), also associated to the three diseases in the main paper, is predicted in this analysis to be associated to asthma and eczema.

CONCLUSIONS

The removal of GWAS-derived association data from our experiment did not substantially alter our findings.
