# Supplementary material for: Computational analysis of multimorbidity between asthma, eczema and rhinitis
Source: PLoS One. 2017 Jun 9;12(6):e0179125. doi: 10.1371/journal.pone.0179125 (PMC5466323; doi:10.1371/journal.pone.0179125)
Supplement: S2 Text — (PDF) [file pone.0179125.s002.pdf]

Text S2. EXTENDED METHODS

Materials and methods

Proteins associated to the multimorbidity of asthma, eczema and rhinitis

Data sources. We built a set of disease-associated genes from the following databases: Online Mendelian Inheritance in Man (OMIM) [1], ENSEMBL 77 Short Variations [2] databases (via BioMart [3]) and the Comparative Toxicogenomics Database (CTD) [4]. The criteria of each database to associate genes to diseases was summarized in the main text, and can be perused in the corresponding references. Naturally, other databases and authors have different criteria when it comes to associating genes to diseases (e.g. type of experimental confirmation, characterization of variations at DNA level, verification of the gene product, etc.). We chose these databases because of their reliability and wide usage in similar studies in recent years [5-10].

Data mining. The process that we followed to harmonize the data from the three databases was: for OMIM, we obtained the common names (HGNC symbols) of disease-associated genes from by parsing the downloadable files available at each website [11]. We chose those diseases with the terms *asthma*, *rhinitis*, *eczema* or *atopic dermatitis* in the TITLE field of the OMIM entries, thus avoiding phenotypes in whose descriptions these terms may appear out of context. The list of resulting diseases are shown in **Table S1**. For CTD, we downloaded the disease-associated genes in common name from [12], selecting those associated to *asthma*, *atopic dermatitis* or *rhinitis*. Only those genes whose association to the disease was attested by experimental means (labeled as *marker*). The diseases For Ensembl Variation, disease-associated genes from Ensembl Variation database were obtained by means of the BioMart web-based tool [13], which allowed us to download the disease-associated variants together with the phenotypes and their corresponding genes in common name. The phenotypes the we selected were *asthma*, *atopic dermatitis* and *rhinitis*.

Data harmonization. Having all three lists of disease-associated genes in the same nomenclature (HGNC symbol), we transformed the gene names to UniProt accession names by means of a Perl script which queried a text version of the UniProt database (for *Homo sapiens* only), downloaded from [14]. Because we base our analysis in the protein interaction network, we considered only those UniProt accessions known to exist at protein level (this information can be found in the *Status* section of all UniProt entries). This resulted in some known disease-associated proteins (like the *PTGDR* gene) being removed from the dataset because their products are only known experimentally at transcript level according to UniProt. The resulting set of disease-associated proteins obtained from each database is shown in **Table S1**. Our criterium was that, once this protein set was buil, any protein not present in it would be assumed to be a novel prediction.

Predicting multimorbidity-associated proteins. The resulting lists of candidate multimorbidity-associated proteins were compared to proteins suggested to be related to multimorbidity in the literature. We chose to use Génie as a validation tool because it does not use a co-occurrence method and outperformed other tools such as PolySearch [15] and Fable [16]. Also, Génie resolves synonyms for gene names, avoiding the ambiguities in the results of similar tools such as Polysearch. As explained in reference [17], Génie software relies on NCBIs curated associations between MedLine records and unambiguous gene identifiers (i.e. genes are not associated to MedLine records statistically but manually), and on a Bayesian classifier to associate them to the user’s query terms. The process is as follows: first, a set of abstracts containing the query terms is used to train a naïve linear Bayesian classifier, which is a weighted list of discriminative words in the selected set of abstracts (compared to the rest of records in MedLine). Then, all abstracts associated to human genes (including manual associations by NCBIs curators) are evaluated by the Bayesian classifier and assigned a *p*-value representing the confidence of the classification. Finally, given a cutoff for abstract selection ($P < 0.01$ by default) a one-sided Fisher's exact test is computed

to define the significance of gene-to-query term relationship, comparing the number of selected abstracts to what is observed in a simulation using a set of 10^4 randomly selected abstracts. The genes are then presented in a list sorted by false discovery rate (FDR, with a cutoff of $FDR < 0.01$ by default). The parameters used to query Génie are available in **Table S4**.

Because PubMed abstracts may contain predicted protein-disease associations, we excluded any abstract containing the words *predicted* or *prediction* to minimize the number of false positives. We also checked that we were not excluding abstracts labelling genes as *predictors* or mentioning genes *with predictive value*, which could have resulted in false negatives. To do so, we simply measured the overlap between the set of PubMed abstracts not containing the words *predicted* nor *prediction* with the set of abstracts containing any of those words plus the words *predictive* or *predictor* (**Table S6**). The overlap between searches shown in **Table S4** and in **Table S6** was zero in all cases, meaning that we were not excluding neither *predictor* genes nor genes with *predictive value* from our searches.

References

1. Amberger JS, Bocchini CA, Schiettecatte F, Scott AF, Hamosh A. OMIM.org: Online Mendelian Inheritance in Man, an online catalog of human genes and genetic disorders. Nucleic Acids Res. 2015; 43: D789-98.
2. Flicek P, Amode MR, Barrell D, Beal K, Billis K, Brent S, *et al*. Ensembl 2014. Nucleic Acids Res. 2014; 42: D749-55.
3. Kasprzyk A. BioMart: driving a paradigm change in biological data management. Database 2011; 2011: bar049
4. Davis AP, Murphy CG, Johnson R, Lay JM, Lennon-Hopkins K, Saraceni-Richards C, *et al*. The Comparative Toxicogenomics Database: update 2013. Nucleic Acids Res 2013; 41: D1104-14.
5. Yang H, Robinson PN, Wang K. Phenolyzer: phenotype-based prioritization of candidate genes for human diseases. Nat Methods. 2015
6. Greene CS, Krishnan A, Wong AK, Ricciotti E, Zelaya RA, Himmelstein DS, Zhang R, Hartmann BM, Zaslavsky E, Sealfon SC, Chasman DI, FitzGerald GA, Dolinski K, Grosser T, Troyanskaya OG. Understanding multicellular function and disease with human tissue-specific networks. Nat Genet. 2015 Jun;47(6):569-76
7. Hamaneh MB, Yu YK. DeCoaD: determining correlations among diseases using protein interaction networks. BMC Res Notes. 2015 Jun 6;8:226
8. Lipner EM, Garcia BJ, Strong M. Network Analysis of Human Genes Influencing Susceptibility to Mycobacterial Infections. PLoS One. 2016 Jan 11;11(1):e0146585.
9. Wang L, Himmelstein DS, Santaniello A, Parvin M, Baranzini SE. iCTNet2: integrating heterogeneous biological interactions to understand complex traits. Version 2. F1000Res. 2015 Aug 5 [revised 2015 Jan 1];4:485
10. Reimand J, Arak T, Adler P, Kolberg L, Reisberg S, Peterson H, Vilo J. g:Profiler-a web server for functional interpretation of gene lists (2016 update). Nucleic Acids Res. 2016 Jul 8;44(W1):W83-9
11. *https://omim.org/downloads/*
12. *http://ctdbase.org/downloads/*
13. *http://www.ensembl.org/biomart/martview/*
14. *ftp://ftp.uniprot.org/pub/databases/uniprot/current_release/knowledgebase/taxonomic_divisions/*
15. Cheng D, Knox C, Young N, Stothard P, Damaraju S, Wishart DS. PolySearch: a web-based text mining system for extracting relationships between human diseases, genes, mutations, drugs and metabolites. Nucleic Acids Res. 2008 Jul 1;36(Web Server issue):W399-405
16. McDonald R, Pereira F. Identifying gene and protein mentions in text using conditional random fields. BMC Bioinformatics. 2005;6 Suppl 1:S6
17. Fontaine JF, Priller F, Barbosa-Silva A, Andrade-Navarro MA. Génie: literature-based gene prioritization at multi genomic scale. Nucleic Acids Res. 2011 Jul;39(Web Server issue):W455-61
