# Supplementary figures and images for: Computational analysis of multimorbidity between asthma, eczema and rhinitis
Source: PLoS One. 2017 Jun 9;12(6):e0179125. doi: 10.1371/journal.pone.0179125 (PMC5466323; doi:10.1371/journal.pone.0179125)

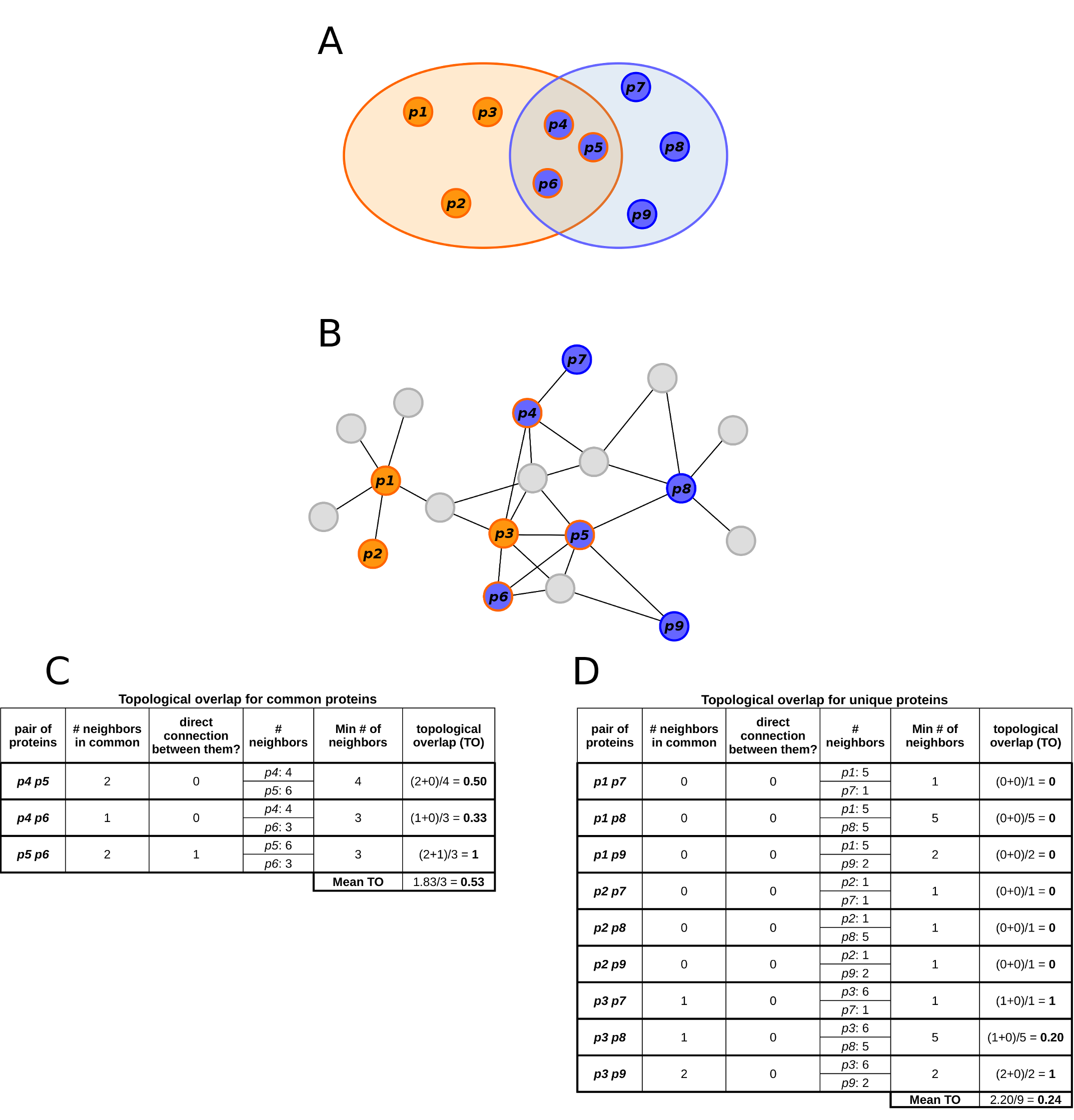

Supplement: S1 Fig — (A) Disease A (orange ellipse) is associated to 6 proteins: p1, p2, p3, p4, p5, p6. Disease B (blue ellipse) is associated to 6 proteins: p4, p5, p6, p7, p8, p9. Three proteins are common to both diseases (p4, p5, p6; shown in the intersection of both diseases, in blue with orange border). (B) Mapping of the disease-associated proteins on to a dummy network. As in the previous figure, common proteins (p4, p5, p6) are shown in blue with orange border. (C) Calculation of the average topological overlap (TO) between the common proteins. (D) Calculation of the average topological overlap between the proteins unique to each disease. (PNG) [file pone.0179125.s003.png]

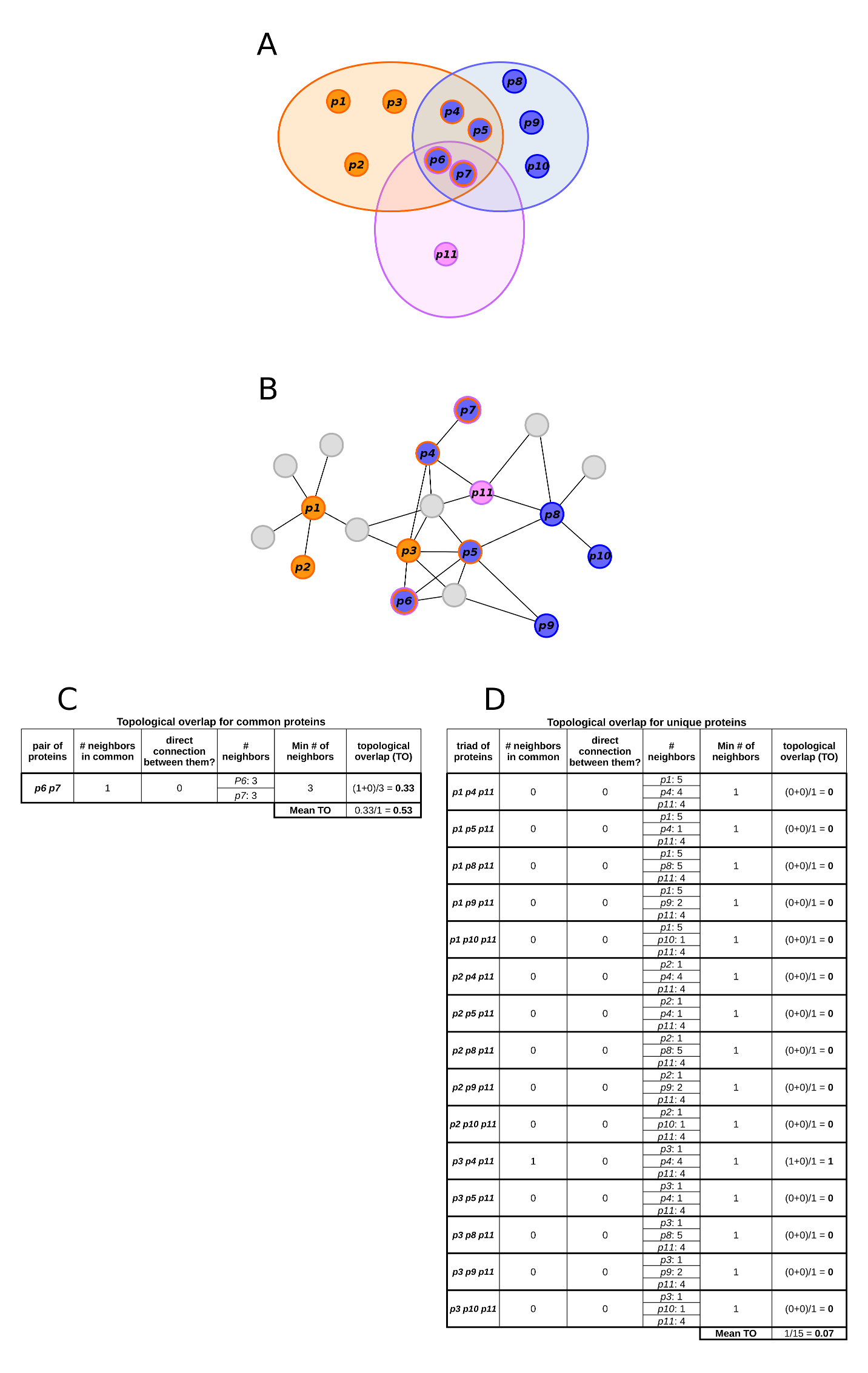

Supplement: S2 Fig — (A) Disease A (orange ellipse) is associated to 7 proteins: p1, p2, p3, p4, p5, p6, p7. Disease B (blue ellipse) is associated to 7 proteins: p4, p5, p6, p7, p8, p9, p10. Disease C (purple ellipse) is associated to 5 proteins: p4, p5, p6, p7, p11. Proteins common to all diseases are p6 and p7 (shown as a blue circles with double purple-orange border). Proteins common solely to diseases A and B are p4 and p5 (shown as blue circles with orange border). There are no proteins common solely to diseases A and C, neither to diseases B and C. (B) Mapping of the disease-associated proteins on to a dummy network. (C) Calculation of the average topological overlap (TO) between the common proteins. (D) Calculation of the average topological overlap between the proteins unique to each disease. (PNG) [file pone.0179125.s004.png]

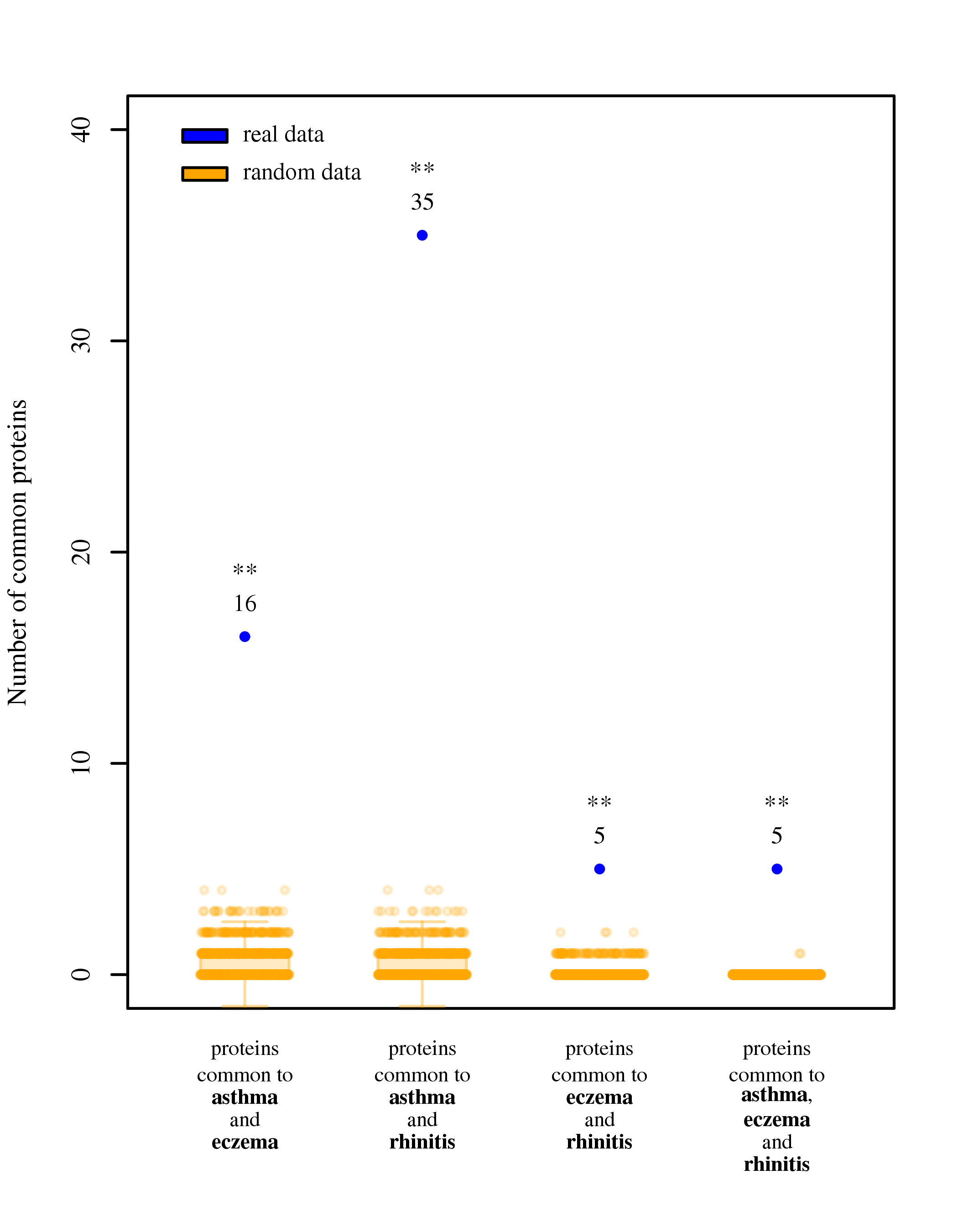

Supplement: S3 Fig — Blue dots indicate the observed fraction of proteins. Orange scatter boxplots indicate random expectation. One asterisk: observed results are significantly larger than random expectation (z-test; P < 0.05). Two asterisks: observed results are significantly larger than random expectation (z-test; P < 0.01). (PNG) [file pone.0179125.s005.png]

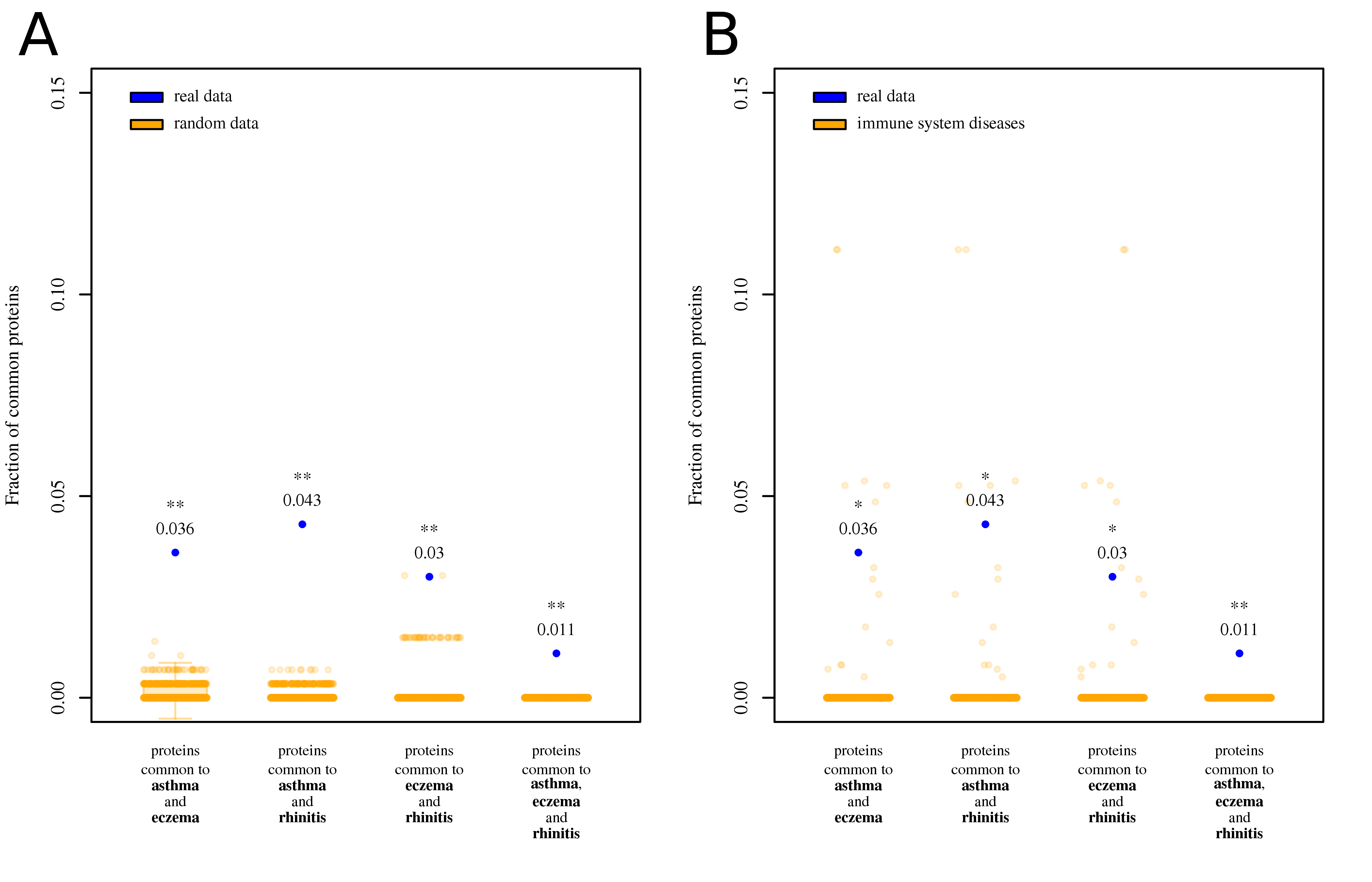

Supplement: S4 Fig — Blue dots indicate the observed fraction of proteins. (A) Orange scatter boxplots indicate random expectation. One asterisk: observed results are significantly larger than random expectation (z-test; P < 0.05). Two asterisks: observed results are significantly larger than random expectation (z-test; P < 0.01). (B) Orange scatter boxplots indicate fraction of associated proteins for pairs/trios of immune system diseases. One asterisk: observed results are significantly larger than random expectation (empirical distribution test; P < 0.05). Two asterisks: observed results are significantly larger than random expectation (empirical distribution test; P < 0.01). (PNG) [file pone.0179125.s006.png]

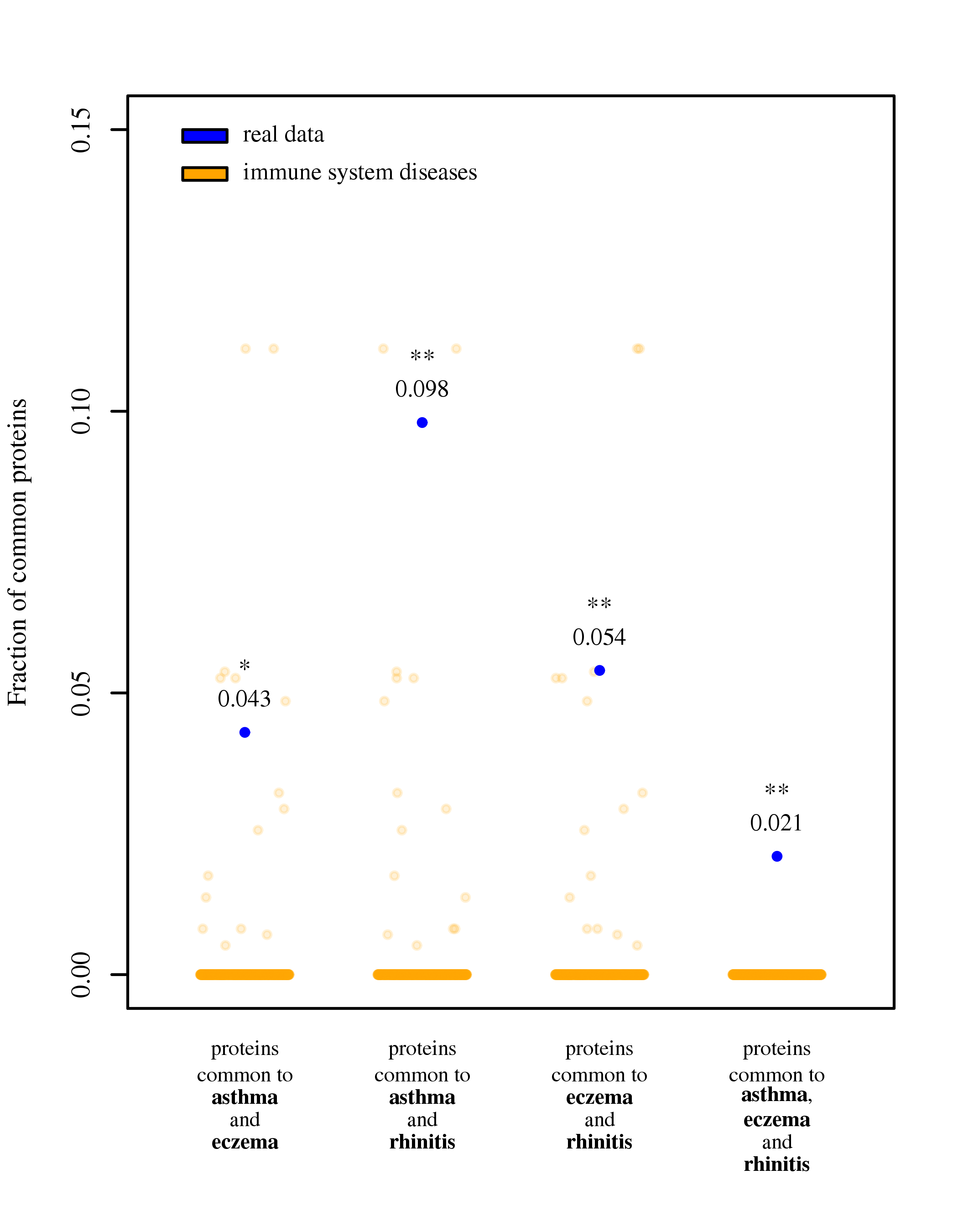

Supplement: S5 Fig — Blue dots indicate the observed fraction of proteins. Orange scatter boxplots indicate fraction of associated proteins for pairs/trios of immune system diseases. One asterisk: observed results are significantly larger than random expectation (empirical distribution test; P < 0.05). Two asterisks: observed results are significantly larger than random expectation (empirical distribution test; P < 0.01). (PNG) [file pone.0179125.s007.png]

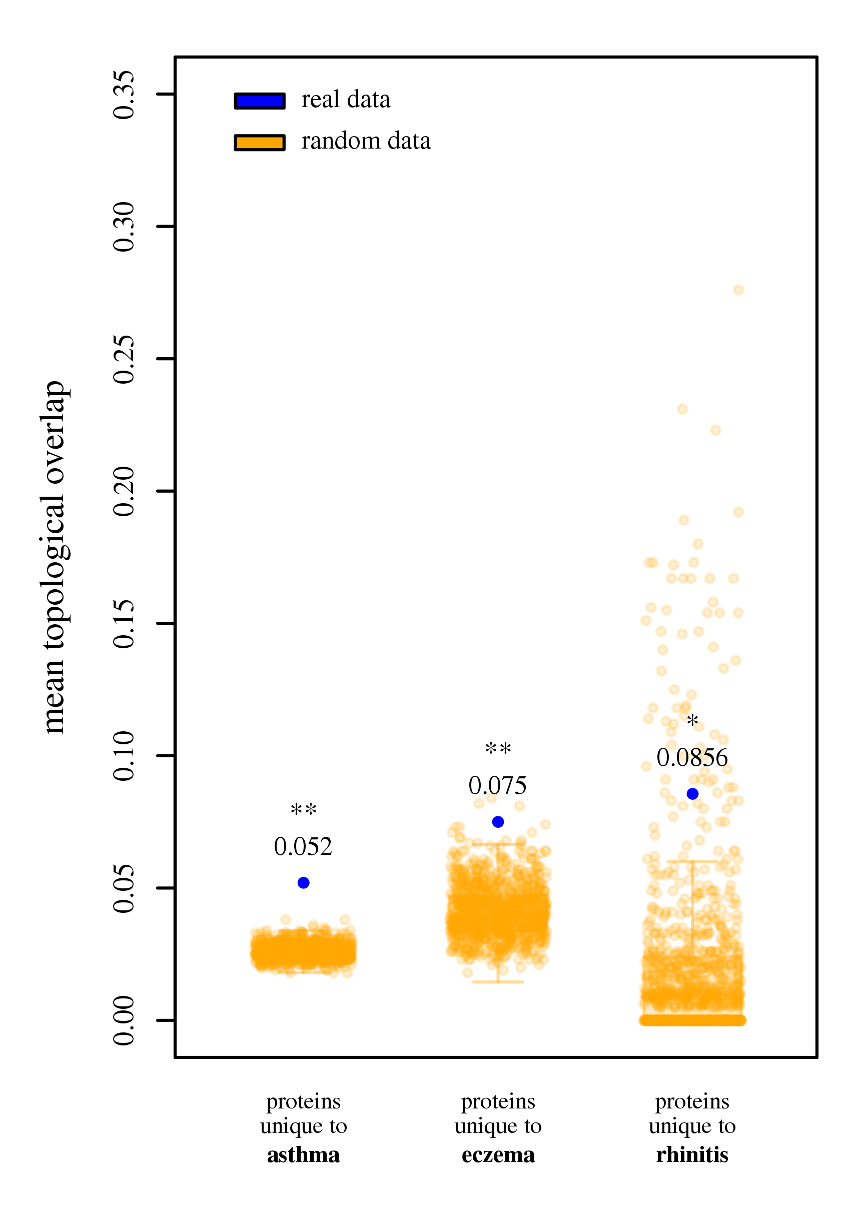

Supplement: S6 Fig — Blue dots indicate the observed mean topological overlap (TO) between proteins uniquely associated to either asthma, eczema or rhinitis. Orange scatter boxplots indicate random expectation. One asterisk: observed results are significantly larger than random expectation (P < 0.05). Two asterisks: observed results are significantly larger than random expectation (P < 0.01). (PNG) [file pone.0179125.s008.png]

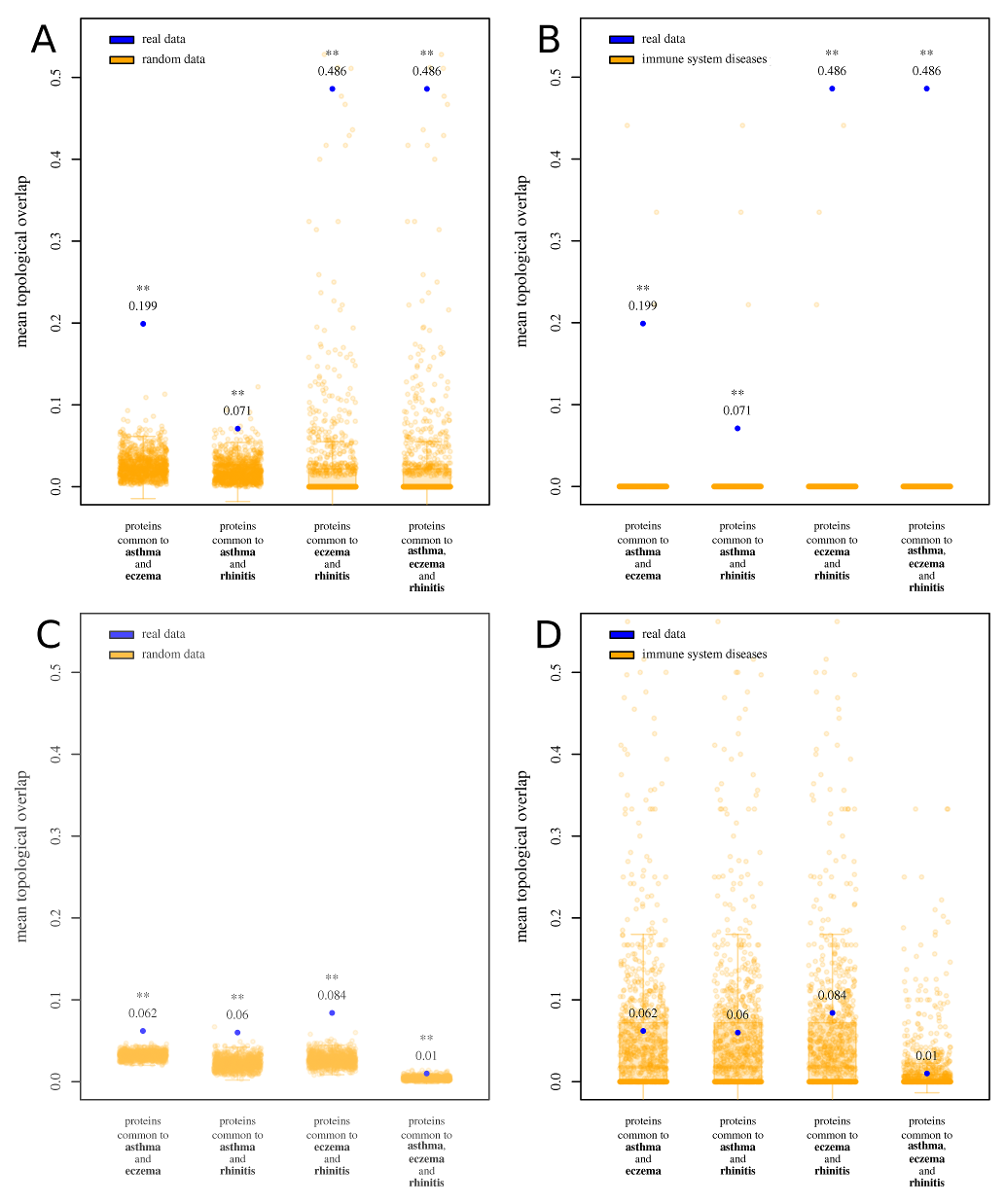

Supplement: S7 Fig — (A) Blue dots indicate the observed mean topological overlap (TO) for proteins common to combinations of asthma, eczema and rhinitis. Orange scatter boxplots indicate random expectation. (B) Blue dots indicate the observed mean TO for proteins common to combinations of asthma, eczema and rhinitis. Orange scatter boxplots indicate observed TO values for pairs/trios of immune system diseases. (C) Blue dots indicate the observed mean TO for proteins unique to combinations of asthma, eczema and rhinitis. Orange scatter boxplots indicate random expectation. (D) Blue dots indicate the observed mean TO for proteins unique to combinations of asthma, eczema and rhinitis. Orange scatter boxplots indicate observed TO values for pairs/trios of immune system diseases. One asterisk: observed results are significantly larger than random expectation (P < 0.05). Two asterisks: observed results are significantly larger than random expectation (P < 0.01). (PNG) [file pone.0179125.s009.png]
