# Supplementary material for: Computational analysis of multimorbidity between asthma, eczema and rhinitis
Source: PLoS One. 2017 Jun 9;12(6):e0179125. doi: 10.1371/journal.pone.0179125 (PMC5466323; doi:10.1371/journal.pone.0179125)
Supplement: S1 Table — OMIM: On-line Mendelian Inheritance in Man; CTD: Comparative Toxicogenomics Database; EV84: Ensembl Variation 84. (PDF) [file pone.0179125.s012.pdf]

Table S1. Source of disease-protein associations. OMIM: On-line Mendelian Inheritance in Man; CTD: Comparative Toxicogenomics Database; EV84: Ensembl Variation 84.

Source	Disease name	Disease name in source / SNP name	Disease-associated protein (UniProt accession)
CTD	asthma	asthma	Q08462; P07550; P05091; P09917; P15514; P05089; P78540; P10415; P02818; P04040; P51671; P13500; P13501; P08571; Q6ZTQ4; Q9UI47; O95715; Q8TE73; P05305; P09488; P09211; P04440; P01909; P01920; P01912; P01911; Q29974; P17693; P09601; P50135; P80365; P05362; Q8IZI9; P29460; P35225; P01584; Q01638; P18510; O95760; P05112; P05113; Q9Y496; P08727; P09238; P14780; Q8TAX7; P10242; P35228; Q6W5P4; P01303; P15559; Q8N138; P09874; Q07343; Q08499; Q9UIL8; Q13093; P00749; P67775; Q6K0P9; Q92878; P12724; P11684; P01137; P35625; P24821; P01375; O75762; Q969D9; P15692; Q8NI36
CTD	asthma	asthma-related traits, susceptibility to, 2	Q6W5P4
CTD	asthma	asthma-related traits, susceptibility to, 5	Q9Y616
CTD	asthma	asthma-related traits, susceptibility to, 7	P36222
CTD	asthma	asthma, aspirin-induced	P51677; Q9Y271; Q9NS75; P04440; Q16873; Q15063; Q9Y5Y4; P43119; P35354; P21731
CTD	dermatitis, atopic	dermatitis, atopic	Q8TC94; Q7Z589; Q9BXL7; Q76M96; P51671; Q92583; O00626; O00175; P13501; P51677; P51681; P02778; P49682; P20930; P16278; P01579; Q8IZI9; P22301; P35225; P01584; O95760; P05112; P05113; P05231; P45983; Q07869; P05109; P16581; Q969D9; O95497; O95498; Q70YC5
CTD	dermatitis, atopic	dermatitis, atopic, 1	P20930
CTD	dermatitis, atopic	dermatitis, atopic, 2	P20930
CTD	dermatitis, atopic	dermatitis, atopic, 4	O14543
CTD	rhinitis	rhinitis	P13569; P50135; P35367; P05112; P01303; P21731; P01282
CTD	rhinitis	rhinitis, allergic, perennial	P02768
CTD	rhinitis	rhinitis, allergic, seasonal	P01037
EV84	asthma	rs10036748	Q15025
EV84	asthma	rs10043228 (GWAS)	Q9Y6G5
EV84	asthma	rs10197862 (GWAS)	Q13478; Q01638
EV84	asthma	rs1042713	P07550
EV84	asthma	rs1042714	P07550
EV84	asthma	rs10512928	Q08462
EV84	asthma	rs1051931	Q13093
EV84	asthma	rs10734479	Q9HCJ2
EV84	asthma	rs10762058 (GWAS)	Q9UI47
EV84	asthma	rs10781329	O14986
EV84	asthma	rs10886289	Q86Y37
EV84	asthma	rs10890784	Q14964
EV84	asthma	rs10970976 (GWAS)	P21399
EV84	asthma	rs11000019 (GWAS)	P07602
EV84	asthma	rs1101999 (GWAS)	Q6K0P9
EV84	asthma	rs11064153	P36941; P37088
EV84	asthma	rs11071559 (GWAS)	P35398
EV84	asthma	rs11558538	P50135
EV84	asthma	rs11696358	Q9ULU4
EV84	asthma	rs121912630	Q9Y616
EV84	asthma	rs12191480	O60242
EV84	asthma	rs12351127	O75899

EV84	asthma	rs12371373	Q14C87
EV84	asthma	rs12379501	P15291
EV84	asthma	rs12450323 (GWAS)	Q9UKT9
EV84	asthma	rs12570188 (GWAS)	Q8WWQ2
EV84	asthma	rs1295686 (GWAS)	P35225
EV84	asthma	rs13361200	Q92608
EV84	asthma	rs13386455	Q53QV2
EV84	asthma	rs13408661 (GWAS)	Q13478; Q01638
EV84	asthma	rs1357099	Q13449
EV84	asthma	rs1361549	Q96IP4
EV84	asthma	rs1367413	P23760
EV84	asthma	rs1422673	Q15025
EV84	asthma	rs1438673 (GWAS)	Q8NI36
EV84	asthma	rs1588265 (GWAS)	Q08499
EV84	asthma	rs16851020	P30542; Q13203
EV84	asthma	rs16925863	P23468
EV84	asthma	rs1701704 (GWAS)	Q9H2S9
EV84	asthma	rs17064520	Q96PU5
EV84	asthma	rs17220663	Q13224
EV84	asthma	rs17294280 (GWAS)	P84022
EV84	asthma	rs17525472 (GWAS)	Q8WXD2
EV84	asthma	rs17774023	Q96BK5
EV84	asthma	rs1805018	Q13093
EV84	asthma	rs1837253 (GWAS)	Q969D9
EV84	asthma	rs1957018	Q13023
EV84	asthma	rs1999716	Q5JR59
EV84	asthma	rs204993 (GWAS)	P40425; Q9Y4H4; Q15109
EV84	asthma	rs20541	P35225
EV84	asthma	rs2058397	Q02930
EV84	asthma	rs2069408 (GWAS)	P61020; P40967; P24941
EV84	asthma	rs2071427	P10827; P20393
EV84	asthma	rs2073643 (GWAS)	O76082
EV84	asthma	rs2183124	P15291
EV84	asthma	rs2225809	P56975
EV84	asthma	rs2233287	Q15025
EV84	asthma	rs2244012 (GWAS)	Q92878
EV84	asthma	rs2267616	Q9HBI1
EV84	asthma	rs2282032	Q9H7Z3
EV84	asthma	rs2284033 (GWAS)	P14784
EV84	asthma	rs229040	Q9UHI8
EV84	asthma	rs2297515	P35228
EV84	asthma	rs2303067	Q9NQ38
EV84	asthma	rs2549003 (GWAS)	P10914
EV84	asthma	rs259892	Q92608
EV84	asthma	rs2632057	Q8IVL1
EV84	asthma	rs2668898	P02794; O76090
EV84	asthma	rs2705520 (GWAS)	Q9NT62
EV84	asthma	rs2786098 (GWAS)	P82279
EV84	asthma	rs3009511	Q96A23

EV84	asthma	rs3019885 (GWAS)	Q8IWU4
EV84	asthma	rs3129890 (GWAS)	P01903
EV84	asthma	rs324981	Q6W5P4
EV84	asthma	rs345065	Q9NS91; P56539
EV84	asthma	rs3730729	Q9UNA4
EV84	asthma	rs3755285	Q9HB29
EV84	asthma	rs3771166 (GWAS)	Q13478
EV84	asthma	rs3771180 (GWAS)	Q13478; Q01638
EV84	asthma	rs3787509	P08579
EV84	asthma	rs3792785	Q15025
EV84	asthma	rs3805236 (GWAS)	Q13480
EV84	asthma	rs3894194 (GWAS)	Q96QA5
EV84	asthma	rs401302	P35443
EV84	asthma	rs404860 (GWAS)	Q99466
EV84	asthma	rs4129267 (GWAS)	P08887
EV84	asthma	rs41364547	P11684
EV84	asthma	rs4673659 (GWAS)	Q15303
EV84	asthma	rs4759966	Q14C87
EV84	asthma	rs4785358	Q2M1K9
EV84	asthma	rs4815617 (GWAS)	Q7Z434
EV84	asthma	rs4833095 (GWAS)	Q15399
EV84	asthma	rs4845783 (GWAS)	Q9UGL9; Q5T5A8
EV84	asthma	rs4950928	P36222
EV84	asthma	rs4950929	P36222
EV84	asthma	rs495198	Q8NEY1
EV84	asthma	rs6094594	O00167
EV84	asthma	rs6436310	P23760
EV84	asthma	rs6485587	O94851
EV84	asthma	rs6571518	Q13023
EV84	asthma	rs6678068	Q9P2M7
EV84	asthma	rs6691378	P36222
EV84	asthma	rs6790467	Q9NS91; P56539
EV84	asthma	rs6790962	Q96A23
EV84	asthma	rs6822478	P53779
EV84	asthma	rs6871536 (GWAS)	Q92878
EV84	asthma	rs6904771	O15217
EV84	asthma	rs6967330 (GWAS)	Q6ZTQ4
EV84	asthma	rs7146136	Q7L622
EV84	asthma	rs7166467	P16452; Q0P6H9
EV84	asthma	rs7212938 (GWAS)	Q96QA5
EV84	asthma	rs7227276	P25391
EV84	asthma	rs727777	Q8TBG9
EV84	asthma	rs7328278 (GWAS)	O15075
EV84	asthma	rs744910 (GWAS)	P84022
EV84	asthma	rs7521681 (GWAS)	Q9UKW4
EV84	asthma	rs7527074 (GWAS)	Q9UBH6
EV84	asthma	rs76043829 (GWAS)	Q9HBL0
EV84	asthma	rs7775228 (GWAS)	P01906; P01909
EV84	asthma	rs7807274 (GWAS)	Q9UL63

EV84	asthma	rs7909484	P56975
EV84	asthma	rs7922491 (GWAS)	Q13976
EV84	asthma	rs8014186	Q9BQT8
EV84	asthma	rs832147	Q13835
EV84	asthma	rs916976	O95714
EV84	asthma	rs9272346 (GWAS)	P01909; Q9GIY3; Q5Y7A7; Q95IE3; P20039; Q30134; P01912; P01906; Q9TQE0; P13761; Q30167; P13760
EV84	asthma	rs9273349 (GWAS)	Q9GIY3; Q5Y7A7; Q95IE3; P20039; Q30134; P01912; P01906; P01920; Q9TQE0; P13761; P01909; Q30167; P13760
EV84	asthma	rs9273373 (GWAS)	Q9GIY3; Q5Y7A7; Q95IE3; P20039; Q30134; P01912; P01906; P01920; Q9TQE0; P13761; P01909; Q30167; P13760
EV84	asthma	rs9275698 (GWAS)	P01906; P01909
EV84	asthma	rs928413 (GWAS)	O95760
EV84	asthma	rs9644708	O95271
EV84	asthma	rs9807989 (GWAS)	Q13478; Q01638
EV84	asthma	rs9823506 (GWAS)	Q7Z7G0
EV84	asthma	rs987870 (GWAS)	P04440; P20036
EV84	asthma	rs9895098 (GWAS)	Q684P5
EV84	dermatitis, atopic	rs10995251 (GWAS)	Q70YC5
EV84	dermatitis, atopic	rs12153855 (GWAS)	Q99941; P22105
EV84	dermatitis, atopic	rs1295686 (GWAS)	P35225
EV84	dermatitis, atopic	rs13015714 (GWAS)	Q13478; Q01638
EV84	dermatitis, atopic	rs1444418 (GWAS)	Q96SZ5
EV84	dermatitis, atopic	rs1665050 (GWAS)	Q6ZNA4
EV84	dermatitis, atopic	rs176095 (GWAS)	P40425; Q9Y4H4; Q99466
EV84	dermatitis, atopic	rs2897442 (GWAS)	Q9Y496
EV84	dermatitis, atopic	rs3091307 (GWAS)	P35225
EV84	dermatitis, atopic	rs3126085 (GWAS)	P20930
EV84	dermatitis, atopic	rs3853601 (GWAS)	Q13838
EV84	dermatitis, atopic	rs4796793 (GWAS)	P40763
EV84	dermatitis, atopic	rs479844 (GWAS)	Q2VPB7
EV84	dermatitis, atopic	rs4821544 (GWAS)	Q15080
EV84	dermatitis, atopic	rs6720763 (GWAS)	A4UGR9
EV84	dermatitis, atopic	rs7613051 (GWAS)	P16278
EV84	rhinitis	rs10197862 (GWAS)	Q13478; Q01638
EV84	rhinitis	rs12450323 (GWAS)	Q9UKT9
EV84	rhinitis	rs1438673 (GWAS)	Q8NI36
EV84	rhinitis	rs17294280 (GWAS)	P84022
EV84	rhinitis	rs1837253 (GWAS)	Q969D9
EV84	rhinitis	rs20541	P35225
EV84	rhinitis	rs4833095 (GWAS)	Q15399
EV84	rhinitis	rs7212938 (GWAS)	Q96QA5
EV84	rhinitis	rs7521681 (GWAS)	Q9UKW4
EV84	rhinitis	rs76043829 (GWAS)	Q9HBL0
EV84	rhinitis	rs9273373 (GWAS)	Q9GIY3; Q5Y7A7; Q95IE3; P20039; Q30134; P01912; P01906; P01920; Q9TQE0; P13761; P01909; Q30167; P13760
OMIM	asthma	ASTHMA-RELATED TRAITS, SUSCEPTIBILITY TO, 2	Q6W5P4
OMIM	asthma	ASTHMA-RELATED TRAITS,	Q9Y616

		SUSCEPTIBILITY TO, 5	
OMIM	asthma	ASTHMA-RELATED TRAITS, SUSCEPTIBILITY TO, 7; ASRT7	P36222
OMIM	asthma	ASTHMA, SUSCEPTIBILITY TO	P09917; P07550; Q8TAX7; P17693; P51671; P50135; P35225; Q13093; P01375; Q9UIL8
OMIM	asthma	IgE RESPONSIVENESS, ATOPIC; IGER	Q9NQ38; Q9UIL8; Q9HBE5; Q96D42; P24394; Q13093; P16109
OMIM	dermatitis, atopic	DERMATITIS, ATOPIC, 2; ATOD2	P20930
OMIM	dermatitis, atopic	WISKOTT-ALDRICH SYNDROME; WAS	P42768
OMIM	rhinitis	ALLERGIC RHINITIS	P35225
OMIM	rhinitis	IgE RESPONSIVENESS, ATOPIC; IGER	Q9NQ38; Q9UIL8; Q9HBE5; Q96D42; P24394; Q13093; P16109
