# Supplementary material for: Computational analysis of multimorbidity between asthma, eczema and rhinitis
Source: PLoS One. 2017 Jun 9;12(6):e0179125. doi: 10.1371/journal.pone.0179125 (PMC5466323; doi:10.1371/journal.pone.0179125)
Supplement: S4 Table — The Génie tool (http://cbdm-01.zdv.uni-mainz.de/~jfontain/cms/) was used to extract gene names present in PubMed abstracts related to a topic of interest, as defined by a PubMed query. (DOC) [file pone.0179125.s015.doc]

**Table S4. Parameters used in the Génie tool.** The Génie tool (*http://cbdm-01.zdv.uni-mainz.de/~jfontain/cms/*) was used to extract gene names present in PubMed abstracts related to a topic of interest, as defined by a PubMed query.

|  | **PubMed query** | **Organism** | **p-value cutoff for abstracts** | **False Discovery**  **Rate for genes** | **number of genes found** |
| --- | --- | --- | --- | --- | --- |
| asthma and eczema | comorbidity AND asthma AND (eczema OR "atopic dermatitis") NOT predicted NOT prediction | *Homo sapiens* | *P* < 0.01 | *FDR* < 0.01 | 413 |
| asthma and rhinitis | comorbidity AND asthma AND rhinitis NOT predicted NOT prediction | *Homo sapiens* | *P* < 0.01 | *FDR* < 0.01 | 369 |
| eczema and rhinitis | comorbidity AND rhinitis AND (eczema OR "atopic dermatitis") NOT predicted NOT prediction | *Homo sapiens* | *P* < 0.01 | *FDR* < 0.01 | 261 |
| asthma, eczema and rhinitis | comorbidity AND asthma AND rhinitis AND (eczema OR "atopic dermatitis") NOT predicted NOT prediction | *Homo sapiens* | *P* < 0.01 | *FDR* < 0.01 | 256 |
