# Supplementary material for: Computational analysis of multimorbidity between asthma, eczema and rhinitis
Source: PLoS One. 2017 Jun 9;12(6):e0179125. doi: 10.1371/journal.pone.0179125 (PMC5466323; doi:10.1371/journal.pone.0179125)
Supplement: S6 Table — The Génie tool was used to extract gene names present in PubMed abstracts related to a topic of interest, as defined by a PubMed query. Unlike S4 Table, this table does not exclude the terms predicted nor prediction, and includes the terms predictive or predictor. (DOC) [file pone.0179125.s017.doc]

**Table S6. Parameters used in the Génie tool.** The Génie tool was used to extract gene names present in PubMed abstracts related to a topic of interest, as defined by a PubMed query. Unlike S4 Table, this table does not exclude the terms *predicted* nor *prediction*, and includes the terms *predictive* or *predictor*.

|  | **PubMed query** | **Organism** | **p-value cutoff for abstracts** | **False Discovery**  **Rate for genes** | **number of abstracts found** | **# abstracts overlapping with S4 Table** |
| --- | --- | --- | --- | --- | --- | --- |
| asthma and eczema | comorbidity AND asthma AND (eczema OR "atopic dermatitis") AND (predictive OR predictor) AND (predicted OR prediction) | *Homo sapiens* | *P* < 0.01 | *FDR* < 0.01 | 1  (PMID: 25725989) | 0 |
| asthma and rhinitis | comorbidity AND asthma AND rhinitis AND (predictive OR predictor) AND (predicted OR prediction) | *Homo sapiens* | *P* < 0.01 | *FDR* < 0.01 | 1  (PMID: 17944984) | 0 |
| eczema and rhinitis | comorbidity AND rhinitis AND (eczema OR "atopic dermatitis") AND (predictive OR predictor) AND (predicted OR prediction) | *Homo sapiens* | *P* < 0.01 | *FDR* < 0.01 | 0 | 0 |
| asthma, eczema and rhinitis | comorbidity AND asthma AND rhinitis AND (eczema OR "atopic dermatitis") AND (predictive OR predictor) AND (predicted OR prediction) | *Homo sapiens* | *P* < 0.01 | *FDR* < 0.01 | 0 | 0 |
