# Supplementary material for: Computational analysis of multimorbidity between asthma, eczema and rhinitis
Source: PLoS One. 2017 Jun 9;12(6):e0179125. doi: 10.1371/journal.pone.0179125 (PMC5466323; doi:10.1371/journal.pone.0179125)
Supplement: S7 Table — Literature predictions were automatically extracted from PubMed abstracts using the Génie data mining tool. The terms used to query PubMed database were those shown in S4 Table minus the word “comorbidity”. Statistical association was calculated by means of a Fisher's Exact Test. Confidence intervals are shown in parentheses. (DOC) [file pone.0179125.s018.doc]

**Table S6. Statistical association between predicted multimorbidity-associated proteins and literature predictions.** Literature predictions were automatically extracted from PubMed abstracts using the Génie data mining tool. The terms used to query PubMed database were those shown in S4 Table minus the word “comorbidity”. Statistical association was calculated by means of a Fisher's Exact Test. Confidence intervals are shown in parentheses.

|  | **association** | |
| --- | --- | --- |
|  | **odds ratio** | ***P*** |
| Literature predictions for **asthma** and **eczema** | 15.44, 95% CI [7.98, 31.02] | 3.15·10^-11^ |
| Literature predictions for **asthma** and **rhinitis** | 4.59, 95% CI [3.03, 6.78] | 2.45·10^-11^ |
| Literature predictions for **eczema** and **rhinitis** | 8.79, 95% CI [2.82, 23.51] | 1.97·10^-4^ |
| Literature predictions for **asthma**, **eczema** and **rhinitis** | 12.58, 95% CI [4.34, 32.57] | 7.89·10^-6^ |
