# Supplementary material for: Computational analysis of multimorbidity between asthma, eczema and rhinitis
Source: PLoS One. 2017 Jun 9;12(6):e0179125. doi: 10.1371/journal.pone.0179125 (PMC5466323; doi:10.1371/journal.pone.0179125)
Supplement: S10 Table — Mean random expectation in shown between parenthesis. The fraction of common edges was calculated using the Jaccard index (see Methods in the main paper). The fraction of common edges is statistically larger than random expectation in all four cases (p-value < 0.01). The fraction of unique edges is statistically lower than random expectation in all four cases (p-value < 0.01). The number of edges associated to asthma: 8560; number of edges associated to eczema: 2584; number of edges associated to rhinitis: 2898. (DOC) [file pone.0179125.s021.doc]

**Table S9 Fraction of edges associated to asthma, eczema and rhinitis**. Mean random expectation in shown between parenthesis. The fraction of common edges was calculated using the Jaccard index (see *Methods* in the main paper). The fraction of common edges is statistically larger than random expectation in all four cases (*p*-value < 0.01). The fraction of unique edges is statistically lower than random expectation in all four cases (*p*-value < 0.01).

|  | **# of common edges** | **Fraction of common edges** | **Fraction of edges unique to asthma** | **Fraction of edges unique to eczema** | **Fraction of edges unique to rhinitis** |
| --- | --- | --- | --- | --- | --- |
| **asthma** and **eczema** | 652 | 0.0621 (rnd:0.0068) | 0.924 (rnd:0.991) | 0.748 (rnd:0.965) |  |
| **asthma** and **rhinitis** | 2741 | 0.0314 (rnd:0.0063) | 0.680 (rnd:0.992) |  | 0.054 (rnd:0.965) |
| **eczema** and **rhinitis** | 173 | 0.0325 (rnd:0.0032) |  | 0.933 (rnd:0.994) | 0.940 (rnd:0.992) |
| **asthma**, **eczema** and **rhinitis** | 172 | 0.0161 (rnd:3.41·10^-6^) | 0.980 (rnd:0.999) | 0.933 (rnd:0.999) | 0.941 (rnd:0.999) |
